# Singlet fission initiating organic photosensitizations

**DOI:** 10.1038/s41598-023-50860-4

**Published:** 2024-01-08

**Authors:** Takao Tsuneda, Tetsuya Taketsugu

**Affiliations:** 1 Department of Chemistry, Faculty of Science, Hokkaido University, Sapporo, 060-0810 Japan; 2 Graduate School of Science Technology and Innovation, Kobe University, Nada-ku, Kobe, Hyogo 657-8501 Japan; 3 Institute for Chemical Reaction Design and Discovery (WPI-ICReDD), Hokkaido University, Sapporo, 001-0021 Japan

**Keywords:** Immunology, Chemistry, Energy science and technology, Materials science, Nanoscience and technology

## Abstract

The feasibility of singlet fission (SF) in organic photosensitizers is investigated through spin-flip long-range corrected time-dependent density functional theory. This study focuses on four major organic photosensitizer molecules: benzophenone, boron-dipyrromethene, methylene blue, and rose bengal. Calculations demonstrate that all these molecules possess moderate $\pi$-stacking energies and closely-lying singlet (S) and quintet (triplet–triplet, TT) excitations, satisfying the essential conditions for SF: (1) Near-degenerate low-lying S and (TT) excitations with a significant S–T energy gap, and (2) Moderate $\pi$-stacking energy of chromophores, slightly higher than solvation energy, enabling dissociation for triplet-state chromophore generation. Moreover, based on the El-Sayed rule, intersystem crossing is found to simultaneously proceed at very slow rates in all these photosensitizers. This is attributed to the fact that the lowest singlet excitation of the monomers partly involves $n\pi ^*$ transitions alongside the main $\pi \pi ^*$ transitions. The proposed mechanisms are strongly substantiated by comparisons with experimental studies.

## Introduction

Photosensitizers find widespread use in various photochemical applications, including photocatalysis, polymer photochemistry, photon upconversion, semiconductors, solar cells, photodynamic therapy, and photoantimicrobials^1,2^. In essence, these molecules transfer energy from incident light to nearby substrates. Photosensitization involves electronic excitation of molecules, leading to two types of energy transfer: direct energy transfer from the photosensitizer (Type I) and indirect energy transfer through the production of singlet oxygen ($^{1}$O$_{2}$) molecules (Type II)^3^. Photosensitizing molecules generally need to possess three key features^2^:

1. Strong absorption of the excitation light.
2. A high yield of intersystem crossing (ISC) to efficiently produce triplet states.
3. A long triplet lifetime to allow for subsequent reactions.

Transition metal complexes are typically not ideal as high-performance photosensitizers due to the inclusion of heavy atoms, which often hinder the fulfillment of the first and third features. Therefore, efforts have been made to develop heavy atom-free photosensitizers. Organic photosensitizers are representative examples of heavy atom-free photosensitizers that exhibit the above three desired features. Examples include benzophenone, BODIPY (boron-dipyrromethene), methylene blue (3,7-bis(di-methylamino) phenothiazin-5-ium chloride), and rose bengal (6-(2,4,5,7-tetraiodo-3-oxo-6-(sodiooxy)-3H-xanthene-9-yl)-2,3,4,5- tetrachlorobenzoic acid). Benzophenone, with a long-lived lowest triplet state at long wavelength less than 320 nm, finds broad application as a photoinitiator. Typically, the photosensitization process of benzophenone involves the photoexcitation to the S$_{1}$ (n$\pi ^*$) state, followed by efficient radiationless relaxation to the T$_2$ excited state of $\pi \pi ^*$ character through a spin-orbit transition^4^. BODIPY offers advantages such as easy availability, high fluorescence quantum yield, low toxicity, and excellent stability, making it suitable for sensors, biology imaging, and solar cells. Through a spin-forbidden transition using the low triplet state, BODIPY-based materials have been reported to transform triplet oxygen molecules into singlet ones^5,6^. The photosensitizability of BODIPY is enhanced by introducing heavy atoms, such as di-halogenation^7^. Methylene blue has three resonance structures that depend on the solvent species and the reactivity of the environment. As a result, the ground state of this molecule exhibits a redox potential that facilitates the oxidation of suitable substrates, such as carbohydrides^8^. In aqueous solution, methylene blue typically exists as a monovalent cation, which causes aggregation and results in a blue shift in the UV–Vis spectra^9^. Rose bengal displays unusual spectroscopic and photochemical properties, including strong absorption in the visible region and a high tendency for ISC to produce a photochemically active triplet excited state. However, at high concentrations ($> 5\times 10^{-5}$) in solution, rose bengal tends to aggregate, which impairs its photochemical response^10^. Despite being organic photosensitizers, these compounds exhibit high photosensitizing abilities attributed to their high ISC rates, which is usually uncommon for heavy atom-free photosensitizers that tend to have low ISC rates.

Singlet fission (SF) is considered a potential mechanism for explaining the spin-forbidden transitions observed in heavy atom-free molecules. SF occurs following singlet electronic excitations, where the singlet (S$_n$, $n\ge$1) state undergoes a spin-allowed transition to the singlet $^1$(TT) excited state, followed by a subsequent transition from the singlet $^1$(TT) to the quintet $^5$(TT) excited states^11–13^:

1 $$\begin{aligned} \text{ S}_0 + \text{ S}_n \rightarrow ^1(\text{ TT})\rightarrow ^5(\text{ TT}) \rightarrow 2\text{ T }, \end{aligned}$$

where (TT) represents the state of two separated triplet states. SF occurs when the triplet (T) excitation energy is slightly lower than half of the singlet excitation energy^11,14^:

2 $$\begin{aligned} (\text{ TT}) \sim 2\Delta E(\text{ T})\lesssim \Delta E(\text{ S}_n), \end{aligned}$$

where $\Delta E$ is the excitation energy. Even without spin-orbit coupling, the spin-forbidden transition from the singlet $^1$(TT) to the quintet $^5$(TT) states rapidly occurs due to the zero-field splitting interaction at the singlet-quintet level-crossing and subsequent re-encounter in the highly disordered region^15^. The quintet state then splits into two T$_1$ states or decays to the ground state^12,13^. Notably, for $\pi$-stacking chromophores like the tetracene dimer, the singlet and quintet states have almost the same energies, with a difference of only about 10 meV^16^. The negligible exchange energy between chromophores allows for considering $^5$(TT) excitations instead of $^1$(TT) excitations in SF discussions. The lifetime of the triplet-state chromophore significantly depends on the concentration of chromophores^17^, as it requires re-encounters of the chromophores in the (TT) states. Therefore, if the conditions of electronic-state energies as expressed in Eq. (2) and the concentration of (TT)-state chromophores are met, the generation of triplet states is expected to proceed through spin-allowed transitions even in heavy atom-free systems.

Recently, we conducted theoretical investigations into the triplet generation mechanisms of 8-*p*-nitrophenyl- and 8-*p*-aminophenyl-tetramethyl BODIPY (TMBODIPY) derivatives, which are heavy atom-free organic photosensitizers. As a result, we proposed that singlet fission (SF) plays a central role in the triplet generation process^18^, which is consistent with experimental femtosecond transient absorption measurements of these derivatives^19^. By observing the correspondence between the solvent dependence of triplet generation rates and the solvent adsorption energies compared to the $\pi$-stacking energies, we performed calculations to obtain the experimental spectra of these derivatives. Utilizing a combination of the spin-flip method and long-range corrected (LC) time-dependent density functional theory (TDDFT), we reached the conclusion that $\pi$-stacking facilitates a spin-allowed transition from the main peak S$_2$ state to the singlet $^1$(TT) state, which subsequently generates triplet states, as shown in Eq. (1). It is worth noting that this process also leads to a slow initiation of intersystem crossings. Based on our study, we proposed that the generation of triplet states in heavy atom-free organic photosensitizers is initiated under two crucial conditions:

1. Near-degenerate low-lying S and (TT) excitations, with a considerable S-T energy gap, and
2. The moderate $\pi$-stacking energy of chromophores, which is higher than but not far from the solvation energy, thus promoting the dissociation that generates triplet-state chromophores.

Therefore, it is essential to explore whether well-known organic photosensitizers satisfy these conditions and, consequently, employ SF as a dominant mechanism in their triplet generation process.

In this study, we investigate the role of SF in the photosensitization process of organic photosensitizers. We examine the excited states of both monomers and $\pi$-stacking dimers of organic photosensitizers, including benzophenone, BODIPY, methylene blue cation, and rose bengal anion molecules in order to elucidate why heavy atom-free organic photosensitizers exhibit a high propensity for generating triplet states despite having low spin-orbit transition rates.

## Computational details

**Figure 1:**
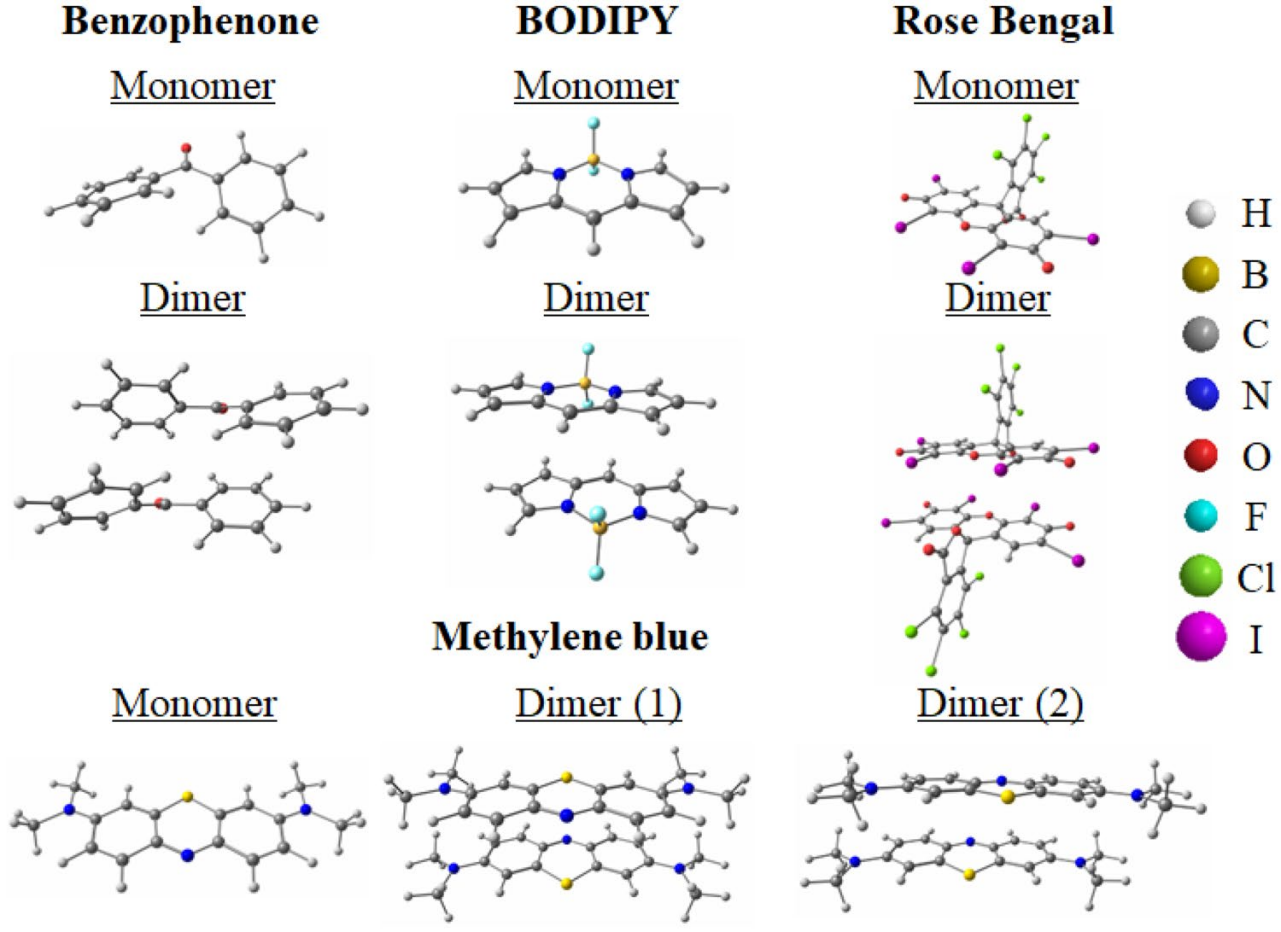
The optimized structures of the calculated systems: the monomers and $\pi$-stacking dimers of benzophenone, BODIPY, methylene blue monovalent cation, and rose bengal divalent anion molecules. For methylene blue, two plausible $\pi$-stacking structures are calculated. The geometry optimizations are performed using LC-BLYP for the monomers and $\omega$B97XD for the dimers with the cc-pVTZ basis set. The Cartesian coordinates of the dimers are compiled in Table S1 of the supporting information.

Collinear spin-flip TDDFT calculations^20,21^ are conducted using the long-range correction (LC)^22,23^ for the Becke 1988 exchange^24^ + Lee–Yang–Parr correlation^25^ (LC-BLYP) functional, along with the $\omega$B97XD^26^ dispersion-corrected LC functional solely for the geometry optimizations of the $\pi$-stacking dimers, employing the cc-pVTZ basis set^27^. To facilitate comparison, we also employ LC-TDDFT^28,29^, utilizing LC-BLYP functional for the monomers and $\omega$B97XD functional for the dimers. The solvent effect is considered by using the conductor-like polarizable continuum model^30^ with isopropyl alcohol and water. It is important to note that spin-flip LC-TDDFT is a highly sophisticated method, known for providing very accurate excitation energies for numerous systems^31,32^, while $\omega$B97XD reliably calculates dispersion forces, including $\pi$-stackings^33^. To our knowledge, spin-flip LC-TDDFT currently stands as the only TDDFT effectively addressing issues encountered in conventional TDDFTs and spin-flip TDDFTs during the calculation of photosensitizer molecules^29^. These issues include the underestimation of charge transfer excitation energies in TDDFTs and overall excitation energies in spin-flip TDDFTs. Note that conventional TDDFTs are also known to underestimate triplet excitation energies and overestimate singlet excitation energies for photosensitizer molecules^34^. Additionally, spin-flip LC-TDDFT proves versatile, readily applicable to systems with a high number of valence electrons, which pose computational challenges for high-level ab initio multireference methods like complete-active-space second-order perturbation theory (CASPT2)^35^. This applicability is illustrated by the investigation of $\pi$-stacking dimers in this study. The optimized structures of the calculated organic photosensitizer molecules for both the monomers and $\pi$-stacking dimers, including benzophenone, BODIPY, methylene blue monovalent cation, and rose bengal divalent anion molecules, are depicted in Fig. 1.

Geometry optimizations are performed for the S$_0$ and T$_1$ excitations of the monomers, and the S$_0$, T$_1$, and $^5$(TT) excitations of the $\pi$-stacking dimers. Several initial $\pi$-stacking structures are examined in the geometry optimizations to identify the most stable structures (see Table S1 of the supporting information for the Cartesian coordinates of the optimized $\pi$-stacking structures). LC-DFT and LC-TDDFT calculations are conducted using the Gaussian 16 Revision A.03 program^36^, while spin-flip LC-TDBLYP calculations are carried out with the development version of the GAMESS-US program^37^, into which we incorporated the spin-flip method for LC-TDDFT.

## Results and discussion

### Electronic excitations of benzophenone

Let us first examine the excitations of benzophenone monomer and $\pi$-stacking dimer. The photosensitization mechanism of benzophenone has been assumed to involve a radiationless relaxation from the S$_1$($n\pi ^*$) state of the monomer to the T$_1$($\pi \pi ^*$) state through a spin-orbit transition to the T$_2$($\pi \pi ^*$) state^38,39^. However, it is worth noting that at a certain concentration, benzophenone tends to form a $\pi$-stacking dimer due to the coordination of isopropyl alcohol and other polar solvent molecules to the oxygen site in the molecular plane (for the $\pi$-stacking geometries, see Fig. S1 of the supporting information). The calculated $\pi$-stacking energy of benzophenone increases by adsorbing isopropyl alcohol molecules from 8.1 to 19.8 kcal/mol. Despite the presence of polar solvent molecules, the moderate $\pi$-stacking energies enable the dimer dissociation even at room temperature. Therefore, benzophenone is expected to undergo SF if it meets the conditions for the excited-state energy levels. Table 1 presents the calculated spin-flip LC-TDBLYP vertical excitation energies, along with the experimental and high-level ab initio complete-active-space second-order perturbation theory (CASPT2) results for comparison. Additionally, it provides the main transitions and oscillator strengths of the corresponding excitations of LC-TDDFTs (LC-TDBLYP and TD$\omega$B97XD) that do not use the spin-flip method. The corresponding excitations of LC-TDDFT to the spin-flip one are determined by comparing the included main transitions. The comparison is conducted in ascending order of excitation energy, as outlined in the subsequent tables. That is, for each spin-flip LC-TDBLYP excitation, we identified and correlated excitations in LC-TDDFT, ensuring that one of the transitions included in the LC-TDDFT excitations corresponds to the main transitions of spin-flip LC-TDBLYP. This correlation is facilitated by exploiting the fact that the reference molecular orbitals of spin-flip LC-TDBLYP are almost the same as those of LC-TDDFT. In Tables S2-1 and S2-2 of the supporting information, the calculated excitation energies of benzophenone have been compiled for a greater number of excitations in ascending order of energy.

**Table 1:** Calculated main vertical excitation energies (eV) of benzophenone molecule for the monomer and $\pi$-stacking dimer by spin-flip LC-TDBLYP with cc-pVTZ basis sets and CPCM solvent effect of isopropyl alcohol.

Excited state	Exp.^a^		Refs.	Spin-flip LC-TDBLYP			LC-TDBLYP/TD$\omega$B97XD			
	eV	nm	eV	eV	nm	Main transitions	No.	eV	nm	*f*
Benzophenone monomer in isopropyl alcohol										
T$_1$			3.33^b^, 3.10^c^	3.20	387			2.93	423	
S$_1$	3.61	343	3.66^b^, 3.64^c^	4.20	295	$$\left\{ \begin{array}{l} \beta {\text{ H }}\rightarrow \beta {\text{ L }} (-\,0.950) \\ \beta {\text{ H }}\rightarrow \beta {\text{ L }}\,+\,7 (0.226) \end{array} \right.$$	1	3.94	315	0.0012
S$_2$	4.40	282	4.33^b^, 4.67^c^	5.02	247	$$\left\{ \begin{array}{l} {\text{ H }}-4\rightarrow \text{ L } (-\,0.780) \\ {\text{ H }}-8\rightarrow \text{ L } (-\,0.460) \end{array} \right.$$	2	5.12	242	0.0314
S$_3$	4.40	282	4.43^b^, 4.67^c^	5.33	233	$$\left\{ \begin{array}{l} {\text{ H }}-3\rightarrow \text{ L } (-\,0.829) \\ {\text{ H }} \rightarrow \text{ L } (-\,0.265) \end{array} \right.$$	3	5.17	240	0.0286
S$_4$	5.00	248	5.39^b^	5.37	231	$$\left\{ \begin{array}{l} {\text{ H }}-2\rightarrow \text{ L } (-\,0.831) \\ {\text{ H }}-1\rightarrow \text{ L } (-\,0.491) \end{array} \right.$$	4	5.32	233	0.5214
Benzophenone $\pi$-stacking dimer in isopropyl alcohol										
T$_1$				3.22	386			2.95	420	
$^5$(TT)$_1$				[6.20]	[200]			5.68	218	
S$_1$	3.61	343		4.21	294	$\beta$H$\rightarrow \beta$L + 1 (− 0.929)	3	5.00	248	0.0096
S$_2$	4.40	282		5.01	247	$$\left\{ \begin{array}{l} {\text{ H }}-9\rightarrow \text{ L } (-\,0.775) \\ {\text{ H }}-18\rightarrow \text{ L } (-\,0.428) \end{array} \right.$$	2	3.99	311	0.0005
S$_3$	5.00	248		5.28	235	$$\left\{ \begin{array}{l} {\text{ H }}-8\rightarrow \text{ L } (-\,0.741) \\ {\text{ H }} \rightarrow \text{ L } (-\,0.317) \end{array} \right.$$	1	3.99	311	0.1232
S$_4$	5.00	248		5.33	233	$$\left\{ \begin{array}{l} {\text{ H }}-6\rightarrow \text{ L } (-\,0.621) \\ {\text{ H }}-3\rightarrow \text{ L } (-\,0.492) \end{array} \right.$$	8	5.21	238	0.5556
S$_8$	6.04	205		6.34	195	$\beta$H$\rightarrow \beta$L + 5 (− 0.936)	16	5.35	232	0.0956

The corresponding excitation energies of LC-TDBLYP (monomer) and TD$\omega$B97XD (dimer) are also listed with the oscillator strengths (*f*) for comparison. Main transitions of spin-flip LC-TDBLYP excitations are also shown with the coefficients of the response functions in parentheses, in which the notations H and L indicate HOMO and LUMO. The presumed quintet (TT) excitation energy is also appended for the dimer in square brackets.

^a^Experimental results in Refs.^40–42^.

^b^CASPT2(16,15) results in Ref.^39^.

^c^CASPT2(12,11) results in Ref.^39^.

As shown in the table, the main peak at 5.00 eV in the UV–Vis spectra of benzophenone is attributed to the S$_4$ excitation of the monomer and the S$_3$ and S$_4$ excitations of the dimer, which exhibit very high oscillator strengths in the corresponding LC-TDBLYP excitations. Conventional theoretical studies have suggested that the photosensitization of benzophenone occurs from the main-peak excitation of the monomer.

**Figure 2:**
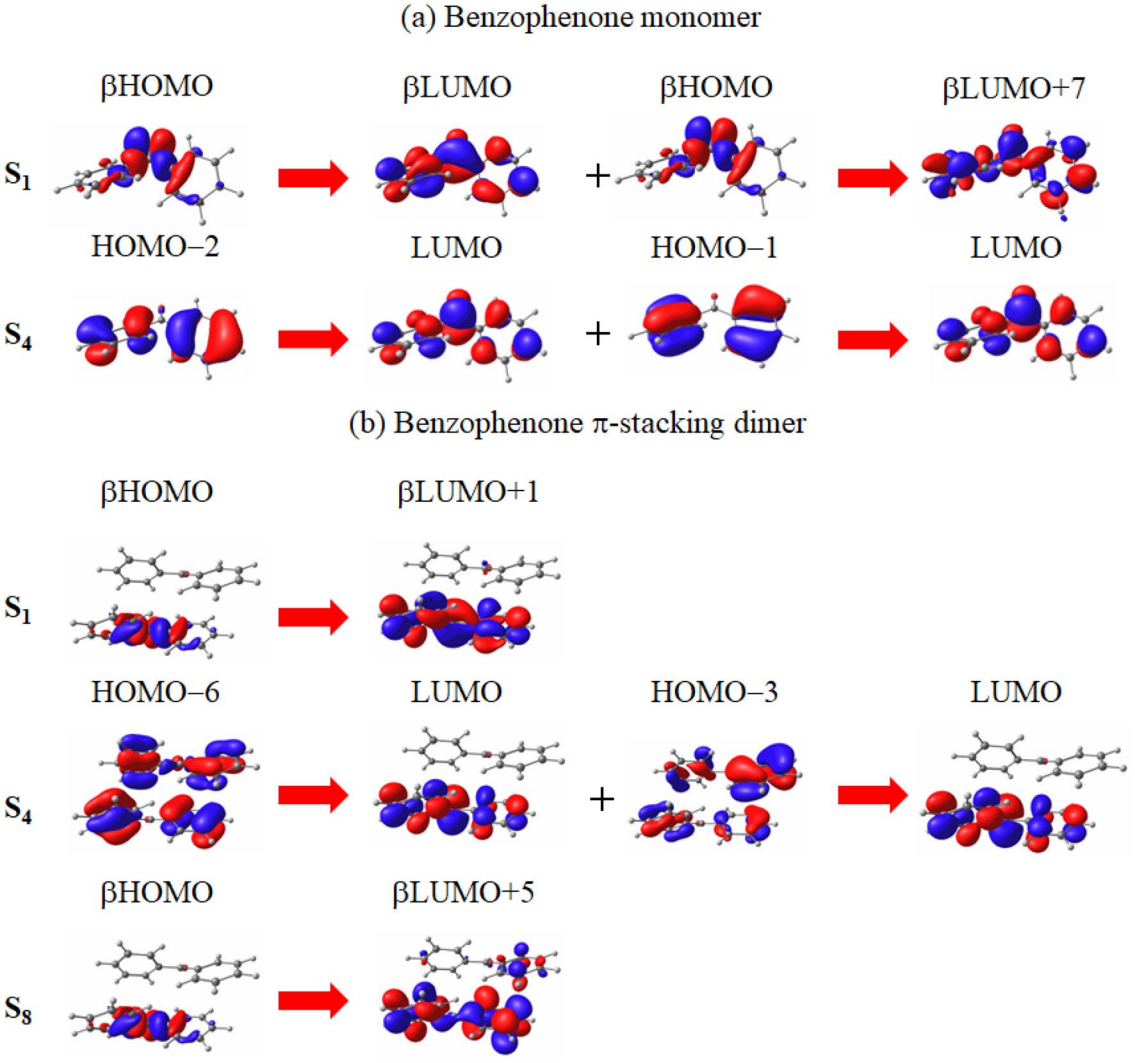
Molecular orbital images corresponding to the main transitions of the S$_1$ and main peak excitations of the (**a**) monomer and (**b**) $\pi$-stacking dimer of benzophenone molecule in isopropyl alcohol solvent. For the monomer and dimer, the main peak excitations are S$_4$ and S$_8$, respectively. The transitions are given in spin-flip LC-TDBLYP/cc-pVTZ calculations, while the molecular orbital images are obtained in LC-BLYP/cc-pVTZ calculations.

Figure 2 illustrates the molecular orbital images corresponding to the main transitions of the S$_1$ and main-peak excitations of the (a) monomer and (b) $\pi$-stacking dimer of the benzophenone molecule in isopropyl alcohol solvent.

For the monomer and dimer, the main peak excitations are S$_4$ and S$_8$, respectively. The transitions are computed using spin-flip LC-TDBLYP/cc-pVTZ calculations, while the molecular orbital images are obtained in LC-BLYP/cc-pVTZ calculations. From the figure, it is evident that the $\beta$HOMO and $\beta$LUMO in the main transition of the S$_1$ excitation closely resemble the natural HOMO and LUMO obtained from the ab initio SA-CASSCF method in a theoretical study^39^. The transition between these molecular orbitals is identified as a $n\pi ^*$ transition. The second main transition also corresponds to a $n\pi ^*$ transition. Considering the small S$_1$–T$_1$ energy gap presented in Table 1, it is anticipated that the S$_1$ excitation will induce ISC in accordance with the El-Sayed rule. This rule suggests that spin-orbit couplings are significant primarily for transitions involving orbitals with different angular momenta, such as $n\pi ^*$ excitations^43^. However, the ISC from the S$_4$ excitation is likely to be slow despite of the $n\pi ^*$ character due to the small induced deviation of the electron distribution and the absence of nearby triplet excitations (For the triplet excitation levels, see Table S1 in the supporting information). Following the S$_4$ excitation, the monomer proceeds with the spin-allowed deexcitations to the S$_1$ state and subsequently initiates the ISC. However, the ISC rate is expected to be small even from the S$_1$ state due to the limited spin-orbit coupling for a small heavy atom-free molecule, as indicated in a conventional theoretical study^44^.

For the dimer, a moderate $\pi$-stacking energy is obtained for benzophenone, fulfilling one of the conditions required for SF to proceed, as mentioned earlier. Consequently, it is crucial to explore whether benzophenone meets the other condition, which involves having near-degenerate low-lying S and (TT) excitations with a considerable S-T energy gap. As seen in Table 1, the S$_8$ excitation, which is assigned to a strong peak in the UV–Vis spectrum, exhibits an excitation energy close to that of the (TT)$_1$ excitation. The former is calculated as 6.34 eV by spin-flip LC-TDBLYP, and the latter is 5.68 eV by TD$\omega$B97XD (although it is estimated to be approximately 6.2 eV by considering the difference between the triplet excitation energies of these methods). This energy gap is, at the very least, at the same level as the S$_1$-T$_1$ energy gap. These results indicate that the $\pi$-stacking dimer of benzophenone satisfies the condition of having near-degenerate low-lying S and (TT) excitations. Upon examining the main transitions of the significant excitations in Fig. 2, it is evident that the S$_1$ excitations in both the monomer and dimer involve intramolecular $n\pi ^*$ transitions. Consequently, ISC also takes place in accordance with the El-Sayed rule. However, it is important to note that the spin-allowed deexcitations to the low singlet states, including the (TT)$_1$ state, are much faster than the spin-forbidden ISCs. Therefore, it is concluded that SF plays a key role in the triplet generation of benzophenone through the $\pi$-stacking under photoirradiation at approximately 6 eV, while ISC also takes place at a slower rate in parallel to SF.

### Electronic excitations of BODIPY

BODIPY is renowned for its role as one of the most prominent photosensitizers, as numerous types of BODIPY derivatives have been developed with diverse characteristics to serve as photosensitizers. In the UV–Vis absorption spectrum of BODIPY, significant peaks appear at approximately 500 nm and 375 nm, for which the intensity of the former peak is 20 times stronger than that of the latter one^45^. BODIPY exhibits very low solubility in polar solvents such as water when hydrophobic groups are not introduced^46^. Despite the introduction of polar groups in the derivatives, the adsorption energies of acetonitrile molecules on tetra-methyl BODIPY derivatives are still too low to exceed the $\pi$-stacking energy^18^. The $\pi$-stacking energy for BODIPY is calculated as 12.3 kcal/mol, facilitating the dissociation process even at room temperature. Consequently, it is believed that BODIPY readily forms the $\pi$-stacking structure and initiates the SF process if the condition for the excited-state energy levels is satisfied.

Table 2 compiles the calculated vertical excitation energies of the BODIPY molecule for both the monomer and $\pi$-stacking dimer.

**Table 2:** Calculated main vertical excitation energies (eV) of BODIPY molecule for the monomer and $\pi$-stacking dimer by spin-flip LC-TDBLYP with cc-pVTZ basis sets and CPCM solvent effect of water.

Excited state	Exp.^a^		Refs.	Spin-flip LC-TDBLYP			LC-TDBLYP/TD$\omega$B97XD			
	eV	nm	eV	eV	nm	Main transitions	No.	eV	nm	*f*
BODIPY monomer in water										
T$_1$			1.86^b^, 1.76^c^, 1.72^d^	1.62	768			1.43	867	
S$_1$	2.48	500	2.48^b^, 2.80^c^, 2.83^d^	2.27	546	$$\left\{ \begin{array}{l} {\text{ H }}\rightarrow \text{ L } (-\,0.697) \\ \beta {\text{ H }}\rightarrow \beta \text{ L } (-\,0.682) \end{array} \right.$$	1	2.93	423	0.6063
T$_2$				3.30	376	$$\left\{ \begin{array}{l} {\text{ H }}-1\rightarrow \text{ L } (0.776) \\ {\text{ H }}\rightarrow \text{ L } (-\,0.472) \end{array} \right.$$	–	–	–	–
S$_2$	3.31	375		3.61	343	H−2$\rightarrow$L (0.926)	3	4.46	278	0.0663
BODIPY $\pi$-stacking dimer in water										
T$_1$				1.63	759			1.43	867	
$^5$(TT)$_1$				[3.10]	[400]			2.72	456	
S$_1$	2.48	500		2.29	542	$$\left\{ \begin{array}{l} {\text{ H }}\rightarrow {\text{ L }}+1 (-\,0.661) \\ {\text{ H }}-1\rightarrow {\text{ L }} (-\,0.660) \end{array} \right.$$	2	3.10	400	1.0845
T$_2$				3.30	376	$$\left\{ \begin{array}{l} {\text{ H }}-4\rightarrow \text{ L } (-0.584) \\ {\text{ H }}-1\rightarrow \text{ L } (-0.485) \end{array} \right.$$	–	–	–	–
S$_2$				3.39	366	$$\left\{ \begin{array}{l} {\text{ H }}\rightarrow \text{ L } (-\,0.763) \\ {\text{ H }}-5\rightarrow \text{ L } (-\,0.440) \end{array} \right.$$	1	2.74	452	0.0000
S$_3$	3.31	375		3.46	358	$$\left\{ \begin{array}{l} {\text{ H }}^2\rightarrow \text{ L}^2 (0.808) \\ \beta {\text{ H }}\rightarrow \beta \text{ L } (0.410) \end{array} \right.$$	4	3.90	318	0.2236

The corresponding excitation energies of LC-TDBLYP (monomer) and TD$\omega$B97XD (dimer) are also listed with the oscillator strengths (*f*) for comparison. Main transitions of spin-flip LC-TDBLYP excitations are also shown with the coefficients of the response functions in parentheses, in which the notations H and L indicate HOMO and LUMO. The presumed quintet (TT) excitation energy is also appended for the dimer in square brackets. Based on the CASPT2 calculation in Ref.^34^, the notations for S$_1$ and T$_2$ excitations are exchanged.

^a^Experimental results in Ref.^45^.

^b^CASPT2(12,11) results in Ref.^34^.

^c^CASSCF results in Ref.^34^.

^d^EOM-CCSD results in Ref.^34^.

**Figure 3:**
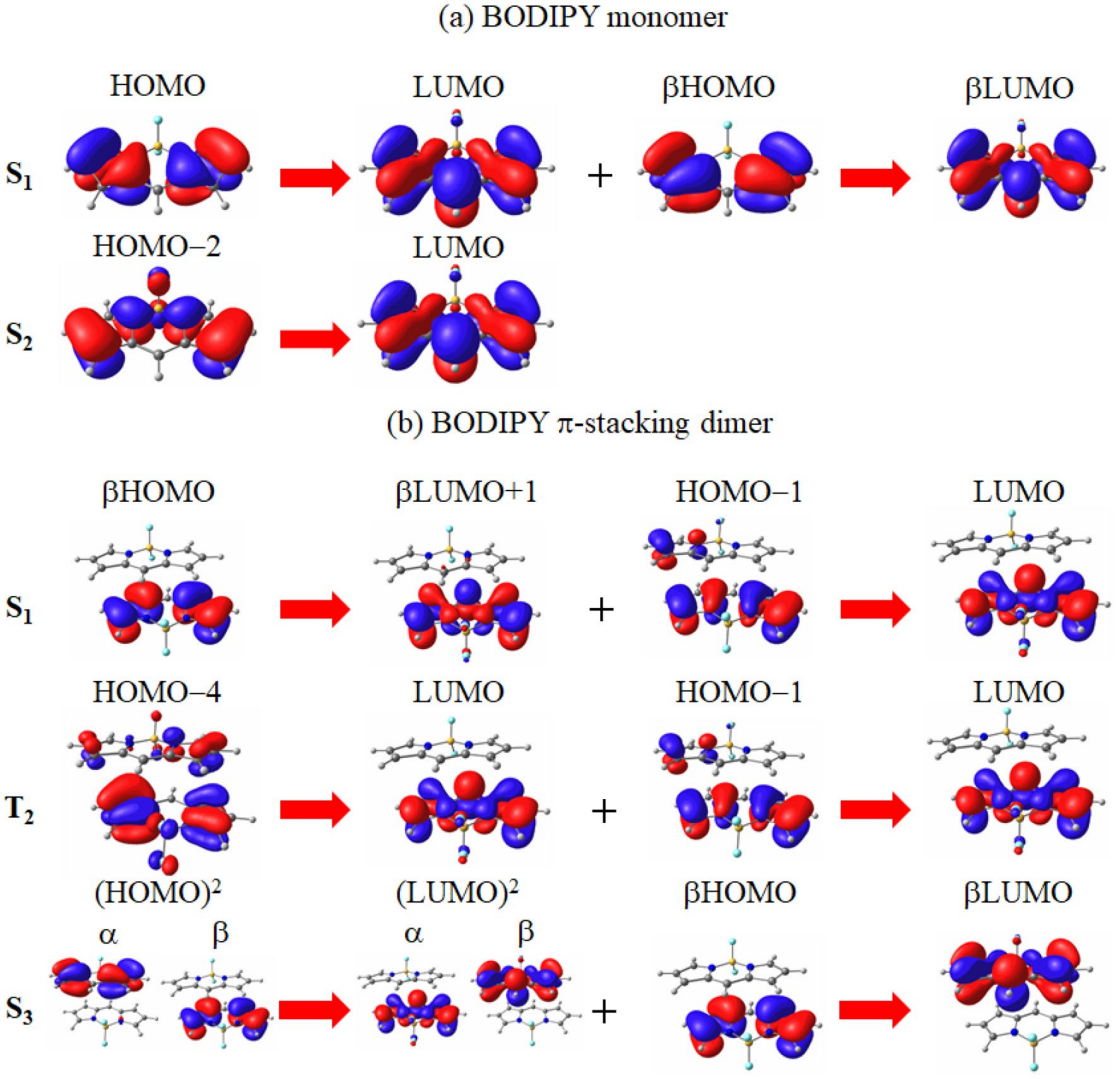
Molecular orbital images corresponding to the main transitions of the S$_1$ and main peak S$_2$ excitations of the (**a**) monomer and (**b**) $\pi$-stacking dimer of BODIPY molecule in water solvent. The transitions are given in spin-flip LC-TDBLYP/cc-pVTZ calculations, while the molecular orbital images are obtained in LC-BLYP/cc-pVTZ calculations.

In Tables S2-3 and S2-4 of the supporting information, the excitation energies of BODIPY have also been organized for additional excitations in ascending order of energy. Note that the notations for S$_1$ and T$_2$ excitations are exchanged based on the molecular orbitals consisting of the main transitions of the monomer in a CASPT2 calculation^34^. The table illustrates the T$_1$–S$_1$ excitation energy gap of the monomer as 0.65 eV for spin-flip LC-TDBLYP, which is considered too large to facilitate a smooth initiation of the spin-forbidden ISC from the S$_1$ excitation. This value is believed to be accurate, as it closely matches the gap between the experimental S$_1$ and CASPT2 T$_1$ excitation energies (0.63 eV). In Fig. 3, the first and second main transitions of the S$_1$ and S$_2$ excitations of spin-flip LC-TDBLYP are depicted.

The figure reveals that the main transitions of the S$_1$ excitation involve the HOMO $\rightarrow$ LUMO transition, which has $\pi \pi ^*$ character. As the T$_1$ excitation, which is the reference of the spin-flip method, consists of $\beta$HOMO$\rightarrow \alpha$LUMO transition, it is suggested that these excitations violate the El-Sayed rule. Considering the large T$_1$–S$_1$ gap and the very strong oscillator strength of the S$_1$ excitation, the ISC from the S$_1$ state is expected to be unfavorable for the monomer.

For the $\pi$-stacking dimer, the S$_1$ excitation of spin-flip LC-TDBLYP corresponds to the main peak based on the calculated oscillator strengths of the corresponding LC-TDBLYP excitation, as shown in Table 2. Although the calculated S$_1$ excitation energy (2.29 eV) closely matches the experimental value (2.48 eV), it is lower than the (TT) excitation energy (2.72 eV for TD$\omega$B97XD). However, it is worth noting that there is another strong peak at 3.31 eV, which is identical as the S$_3$ excitation with a strong oscillator strength. Comparing this to the S$_3$ excitation energy of spin-flip LC-TDBLYP (3.46 eV), the (TT)$_1$ excitation energy of $\omega$B97XD, 2.72 eV, appears considerably low. Nevertheless, when considering the 0.2 eV difference between the calculated T$_1$ excitation energies of spin-flip LC-TDBLYP and $\omega$B97XD, this energy gap decreases to only 0.34 eV, almost half of the S$_1$–T$_1$ gap (0.66 eV). Considering that the transition from the S$_3$ to (TT)$_1$ states is spin-allowed, SF is expected to proceed through this transition. This is supported by the main transitions of the S$_1$ excitation of the dimer in Fig. 3, where the main transitions predominantly exhibit $\pi \pi ^*$ characters, indicating a small ISC rate for the $\pi$-stacking dimer. Similar to the monomer, the oscillator strength of the S$_1$ excitation is very large for the $\pi$-stacking dimer, implying very fast deexcitations. Therefore, it is concluded that SF primarily generates triplet states for BODIPY.

### Electronic excitations of methylene blue monovalent cation

For methylene blue, the monovalent cation is the prevalent state in water solvent, and its aggregation results in a blue shift in UV–Vis spectra^9^. Methylene blue is believed to initiate a proton-coupled electron transfer, which is dependent on the solvent species and the reactivity of the environment^8^. Conventional theoretical studies utilizing conventional TDDFT calculations have assumed that the triplet generation of the methylene blue monomer proceeds through a vibronic ISC from S$_1$($\pi \pi ^*$) to T$_2$($\pi \pi ^*$) states^47^. However, this mechanism, which violates the El-Sayed rule, does not explain the cause for the experimentally-observed active triplet-generation. Moreover, it has been reported that conventional TDDFTs using pure and global hybrid functionals fail to reproduce the spectral features of methylene blue due to limitations in describing the excitations of $\pi$-conjugated systems^48^. Additionally, an experimental Fourier-transform infrared spectrum study^9^ concludes that the methylene blue monovalent cation forms the $\pi$-stacking dimer in water solvent. Consequently, it is essential to determine whether the methylene blue cation satisfies the condition for the excited-state energy levels.

Table 3 summarizes the calculated vertical excitation energies of the methylene blue monovalent cation for both the monomer and $\pi$-stacking dimer.

**Table 3:** Calculated vertical excitation energies (eV) of methylene blue monovalent cation molecule for the monomer and $\pi$-stacking dimer by spin-flip LC-TDBLYP, LC-TDBLYP (monomer) and TD$\omega$B97XD (dimer) with cc-pVTZ basis sets and CPCM solvent effect of water.

Excited state	Exp.		Ref.^c^	Spin-flip LC-TDBLYP			LC-TDBLYP/TD$\omega$B97XD			
	eV	nm	eV	eV	nm	Main transitions	No.	eV	nm	*f*
Methylene blue monomer in water										
T$_1$			1.63	1.21	1021			1.16	1066	
S$_1$	1.87^a^	662	2.09	1.78	698	$$\left\{ \begin{array}{l} \beta {\text{ H }}\rightarrow \beta \text{ L } (-\,0.705) \\ {\text{ H }}\rightarrow \text{ L } (-\,0.682) \end{array} \right.$$	1	2.45	506	1.1577
T$_2$				2.59	478	$$\left\{ \begin{array}{l} {\text{ H }}-1\rightarrow \text{ L } (0.948) \\ {\text{ H }}^2\rightarrow \text{ L}^2 (-\,0.207) \end{array} \right.$$	–	–	–	–
S$_2$				3.10	399	$$\left\{ \begin{array}{l} \beta {\text{ H }}\rightarrow \beta \text{ L } (-\,0.645) \\ {\text{ H }}\rightarrow \text{ L } (0.644) \end{array} \right.$$	–	–	–	
Methylene blue $\pi$-stacking dimer (1) in water										
T$_1$				1.21	1021			1.54	805	
$^5$(TT)$_1$	2.05^b^	606		[2.04]	[609]			2.59	478	
S$_1$				1.76	705	$$\left\{ \begin{array}{l} {\text{ H }}\rightarrow {\text{ L }}+1 (-\,0.676) \\ {\text{ H }}-3\rightarrow {\text{ L }} (-\,0.524) \end{array} \right.$$	3	2.79	445	0.1191
T$_2$				2.54	489	$$\left\{ \begin{array}{l} {\text{ H }}-2\rightarrow \text{ L } (-\,0.736) \\ {\text{ H }}-3\rightarrow \text{ L } (-\,0.524) \end{array} \right.$$	–	–	–	–
S$_2$				2.81	442	$$\left\{ \begin{array}{l} {\text{ H }}\rightarrow \text{ L } (-\,0.697) \\ \beta {\text{ H }}\rightarrow \beta \text{ L } (-\,0.615) \end{array} \right.$$	1	2.17	571	0.0000
Methylene blue $\pi$-stacking dimer (2) in water										
T$_1$				1.23	1004			1.44	861	
$^5$(TT)$_1$	2.05^b^	606		[2.12]	[583]			2.49	497	
S$_1$				1.79	694	$$\left\{ \begin{array}{l} {\text{ H }}\rightarrow {\text{ L }}+1 (-\,0.695) \\ {\text{ H }}-2\rightarrow \text{ L } (-\,0.600) \end{array} \right.$$	1	2.14	580	0.0017
T$_2$				2.58	481	$$\left\{ \begin{array}{l} {\text{ H }}-3\rightarrow \text{ L } (-\,0.819) \\ {\text{ H }}-2\rightarrow \text{ L } (-\,0.398) \end{array} \right.$$	–	–	–	–
T$_3$				2.64	470	$$\left\{ \begin{array}{l} \beta {\text{ H }}\rightarrow \beta \text{ L } (-\,0.758) \\ {\text{ H }}^2\rightarrow \text{ L}^2 (-\,0.611) \end{array} \right.$$	–	–	–	–
S$_2$	2.38^b^	520		2.84	437	H$\rightarrow$L (0.930)	2	2.48	499	1.8153

Main transitions of spin-flip LC-TDBLYP excitations are also shown with the coefficients of the response functions in parentheses, in which the notations H and L indicate HOMO and LUMO. The oscillator strengths (*f*) of LC-TDBLYP (monomer) and TD$\omega$B97XD (dimer) are also listed. The presumed quintet (TT) excitation energy is also appended for the dimer in square brackets. Based on the CASPT2 calculation in Ref.^47^, the notations for S$_1$ and T$_2$ excitations are exchanged.

^a^Experimental results in Ref.^9^.

^b^Experimental results in Ref.^49^.

^c^CASPT2(14,12) results in Ref.^47^.

In Tables S2-5 and S2-6 of the supporting information, the excitation energies of methylene blue monovalent cation have also been displayed in ascending order of energy for additional excitations. In the table, the S$_1$–T$_1$ energy gap of the monomer decreases from 1.29 eV (LC-TDBLYP) to 0.57 eV (spin-flip LC-TDBLYP), depending on the double excitation effect introduced by the spin-flip method. Although the energy gap approaches closely to the value of the reference CASPT2 (0.46 eV), it is still considered large for progressing ISC. Note that, according to the results of CASPT2, the energy relationship between the S$_1$ and T$_2$ excitations is inverted, and their energy gap is very small. This implies that ISC is likely to occur if the main transitions of each excitation satisfy the El-Sayed rule.

**Figure 4:**
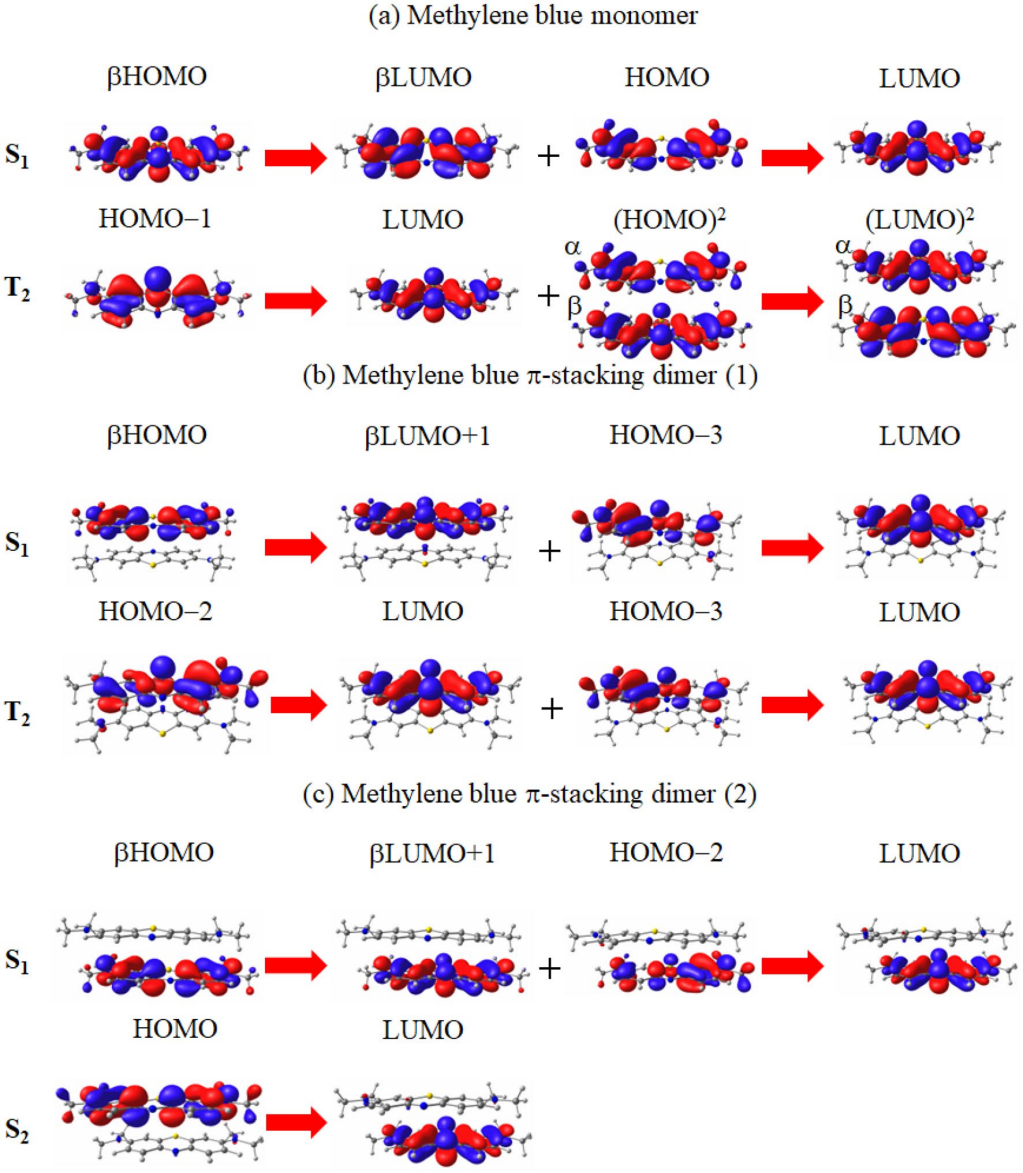
Molecular orbital images corresponding to the main transitions of the S$_1$ and T$_2$ excitations of the (**a**) monomer and (**b**) $\pi$-stacking dimer (1), and (**c**) $\pi$-stacking dimer (2) of methylene blue monovalent cation molecule in water solvent. The transitions are given in spin-flip LC-TDBLYP/cc-pVTZ calculations, while the molecular orbital images are obtained in LC-BLYP/cc-pVTZ calculations.

Figure 4 illustrates the molecular orbitals corresponding to the main transitions of the S$_1$ and T$_2$ excitations, which are the lowest-lying excitations in the spin-flip LC-TDBLYP calculation.

The figure shows that both the S$_1$ and T$_2$ excitations of the monomer mainly consists of $\pi \pi ^*$ transitions, which violate the El-Sayed rule. However, the figure also shows that the main transitions of the S$_1$ excitation minimally incorporate shifts between $\pi$ orbitals and the non-bonding orbitals of boron or nitrogen. Therefore, spin-orbit coupling is extremely small but not entirely absent. Considering the substantial oscillator strength, this implies that ISC is likely to occur at a very slow rate for the monomer of the methylene blue monovalent cation.

For the $\pi$-stacking dimer, there are two possible structures, stacked in opposite (1) and same (2) molecular orientations (for the optimized structures, see Fig. 1 and Table S1 in the supporting information). It is noteworthy that an increase in concentration results in the appearance of a peak, likely arising from dimerization, with a shorter wavelength (606 nm) than that of the main peak^49^, corresponding to the peak of $^1$(TT)$_1$ excitation. Contrary to intuition, substantial calculated $\pi$-stacking energies are observed for this cation in dimers (1) and (2): 19.8 and 19.4 kcal/mol, respectively. These moderate $\pi$-stacking energies are appropriate for both dimer formation in water and dissociation at room temperature. Table 3 compiles the calculated excitation energies of the dimers. As shown in the table, no peaks is generated around the peak of the (TT) excitation, 609 and 583 nm for dimers (1) and (2), respectively, in the spin-flip LC-TDBLYP calculations, which are close to the new peak at 606 nm. On the other hand, a peak at 2.04 eV emerges upon the dimer formation in the experimental UV–Vis spectrum^9^. As the calculated singlet excitations of the dimer do not reveal any newly appearing excitations except for the (TT) ones in the spin-flip LC-TDBLYP calculations, we assign this peak as the (TT) excitations. Consequently, we consider that SF rapidly proceeds through this singlet (TT) excitations for both these dimers.

Figure 4 displays the main transitions for the S$_1$ and T$_2$ excitations of the dimers. As shown in the figure, the main transitions of both the S$_1$ and T$_2$ excitations fundamentally possess intramolecular $\pi \pi ^*$ characters. However, similar to the excitations of the monomer, there are slight $n\pi ^*$ transitions included. ISC is, therefore, considered to proceed slowly in the presence of a triplet state near the S$_1$ excitation. According to Table 3, the excitation energies of S$_1$ and T$_2$ are very close to those of the monomer. These results support that ISC may also occur very slowly in the $\pi$-stacking dimer. Nonetheless, as mentioned earlier, due to the presumed absorption of the (TT) excitation, observed in the UV–Vis spectrum, SF would preferentially proceed in triplet generations.

### Electronic excitations of rose bengal divalent anion

Rose bengal, an organic photosensitizer containing heavy iodine groups, is well-known for its strong absorption band in the 500–570 nm region^10^ with a high ISC rate^50^, facilitating the generation of triplet states. In solution, rose bengal tends to aggregate at high concentrations, leading to a reduction in photochemical response^10^. Only a few theoretical studies have been reported so far on the triplet generation of rose bengal, assuming that it proceeds through ISC. According to these studies, the S$_1$–T$_1$ energy gap is evaluated to be large, approximately 1 eV, and the main transition of the S$_1$ excitation is a $\pi \pi ^*$ transition, violating the El-Sayed rule^51^. However, it is important to note that a considerable $\pi$-stacking energy of 14.4 kcal/mol is unexpectedly obtained for the rose bengal divalent anion. This energy is adequate for stacking and facilitates the dissociation of such anions at room temperature. This satisfies one of the conditions for SF to proceed. Therefore, exploring the contribution of SF, despite its heavy atom-containing structure, is significant for understanding the triplet generation of rose bengal.

Table 4 compiles the calculated excitation energies of the rose bengal divalent anion monomer.

**Table 4:** Calculated vertical excitation energies (eV) of rose bengal divalent anion monomer by spin-flip LC-TDBLYP and LC-TDBLYP with cc-pVTZ basis sets except for iodine, LanL2DZ basis set for iodine, and CPCM solvent effect of water.

Excited state	Exp.^a^		Spin-flip LC-TDBLYP			LC-TDBLYP			
	eV	nm	eV	nm	Main transitions	No.	eV	nm	*f*
T$_1$			0.89	1401			2.48	500	
S$_1$			1.45	856	$$\left\{ \begin{array}{l} {\text{ H }}\rightarrow {\text{ L }}+2 (-\,0.723) \\ {\text{ H }}\rightarrow {\text{ L }}+1 (-\,0.621) \end{array} \right.$$	7	4.74	261	0.0057
S$_2$			1.68	739	$$\left\{ \begin{array}{l} {\text{ H }}\rightarrow \text{ L+3 } (-\,0.792) \\ {\text{ H }}\rightarrow \text{ L+5 } (-\,0.413) \end{array} \right.$$	3	4.28	290	0.0215
S$_3$			1.74	714	$$\left\{ \begin{array}{l} {\text{ H }}-3\rightarrow \text{ L } (-\,0.884) \\ {\text{ H }}-15\rightarrow \text{ L } (-\,0.194) \end{array} \right.$$	12	5.19	239	0.0365
S$_4$	2.27	546	2.30	539	$$\left\{ \begin{array}{l} {\text{ H }}-5\rightarrow \text{ L } (-\,0.616) \\ {\text{ H }}-1\rightarrow \text{ L } (-\,0.540) \end{array} \right.$$	2	4.21	294	0.0788
S$_5$	2.41	515	2.72	456	$$\left\{ \begin{array}{l} {\text{ H }}-16\rightarrow \text{ L } (-\,0.742) \\ {\text{ H }}-10\rightarrow \text{ L } (-\,0.532) \end{array} \right.$$	–	–	–	–
S$_6$	2.54	488	2.77	447	$$\left\{ \begin{array}{l} \beta {\text{ H }}\rightarrow \beta \text{ L } (0.930) \\ {\text{ H }}-5\rightarrow \text{ L } (-\,0.1755) \end{array} \right.$$	1	4.14	300	0.0714
S$_7$			2.97	417	$$\left\{ \begin{array}{l} {\text{ H }}\rightarrow \text{ L+7 } (0.838) \\ {\text{ H }}\rightarrow \text{ L+2 } (0.271) \end{array} \right.$$	–	–	–	–

Main transitions of spin-flip LC-TDBLYP excitations are also shown with the coefficients of the response functions in parentheses, in which the notations H and L indicate HOMO and LUMO. The oscillator strengths (*f*) of LC-TDBLYP are also listed.

^a^Experimental results in Ref.^52^.

In Tables S2-7 and S2-8 of the supporting information, the excitation energies for the divalent anion of rose bengal have also been arranged in ascending order of energy for additional excitations. As shown in the table, introducing the spin-flip method dramatically decreases the LC-TDBLYP excitation energies to about half of their values, bringing them close to the experimental ones. This indicates that the double excitation effect plays a main role in the singlet excitations of rose bengal. Therefore, the correspondence between the excitations of spin-flip LC-TDBLYP and LC-TDBLYP is questionable. It is, however, observed that the excitation energies of spin-flip LC-TDBLYP corresponding to excitations with strong oscillator strengths are in good agreement with the experimental peak energies. Furthermore, this decreases the S$_1$–T$_1$ energy gap from 1.66 eV in LC-TDBLYP to 0.56 eV in spin-flip LC-TDBLYP.

**Figure 5:**
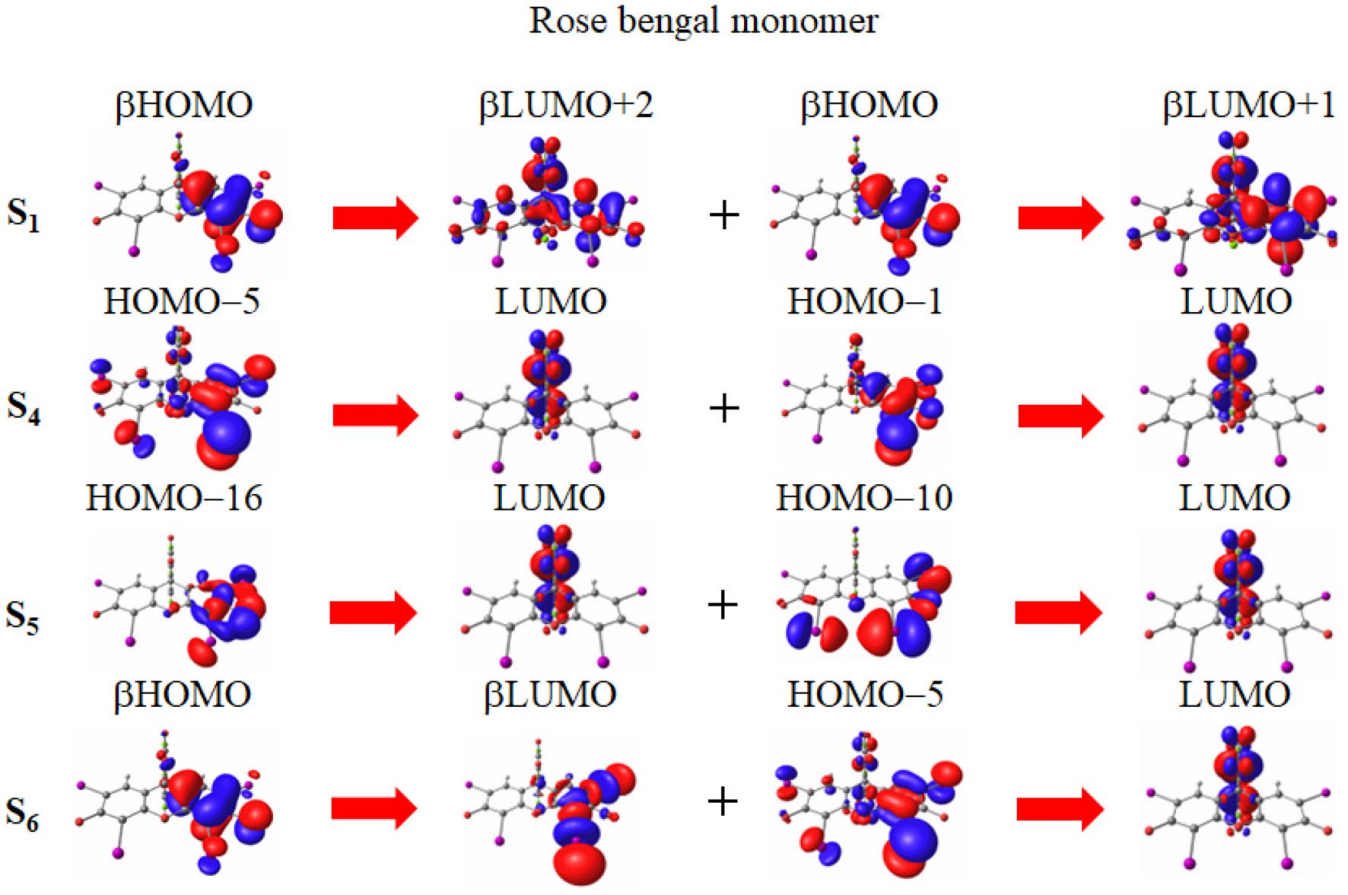
Molecular orbital images corresponding to the main transitions of the S$_1$ and main peak S$_4$, S$_5$, and S$_6$ excitations of the monomer of rose bengal divalent anion molecule in water solvent. The transitions are given in spin-flip LC-TDBLYP/cc-pVTZ calculations, while the molecular orbital images are obtained in LC-BLYP/cc-pVTZ calculations.

In Fig. 5, the main transitions of the S$_1$ and main-peak S$_4$, S$_5$, and S$_6$ excitations of the rose bengal monomer are depicted.

The figure clearly shows that almost all of these excitations have charge transfer characteristics. In particular, since the S$_1$ excitation corresponds to long-range charge transfers from the left to the right side of rose bengal, it is presumed to possess large spin–orbit couplings with the triplet states. Furthermore, this excitation involves a $n\pi ^*$ transition from the $\pi$ orbital to the *n* orbital of iodine. This suggests that ISC is relatively more significant compared to the other three molecules. However, the LC-TDBLYP calculations do not provide closely-lying triplet excitations for the S$_1$ and other low-lying singlet excitations (for the triplet excitation energies, see Table S3 in the supporting information). It is, therefore, presumed that the spin-orbit transition slowly proceeds for the monomer.

Table 5 compiles the calculated excitation energies of the rose bengal divalent anion $\pi$-stacking dimer.

**Table 5:** Calculated vertical excitation energies (eV) of rose bengal divalent anion $\pi$-stacking dimer by spin-flip LC-TDBLYP and TD$\omega$B97XD with cc-pVTZ basis sets except for iodine, LanL2DZ basis set for iodine, and CPCM solvent effect of water.

Excited state	Exp.^a^		Spin-flip LC-TDBLYP			TD$\omega$B97XD			
	eV	nm	eV	nm	Main transitions	No.	eV	nm	*f*
T$_1$			0.67	1859			2.16	575	
(TT)$_1$			[1.34]	[928]			4.31	288	
S$_1$			1.23	1004	$$\left\{ \begin{array}{l} {\text{ H }}\rightarrow \text{ L+4 } (0.812) \\ {\text{ H }}\rightarrow \text{ L+5 } (-\,0.548) \end{array} \right.$$	11	4.29	289	0.0000
S$_2$			1.40	883	$$\left\{ \begin{array}{l} {\text{ H }}\rightarrow \text{ L+5 } (-\,0.689) \\ {\text{ H }}\rightarrow \text{ L+6 } (-\,0.369) \end{array} \right.$$	6	3.95	314	0.0014
S$_3$			1.51	819	$$\left\{ \begin{array}{l} {\text{ H }}-9\rightarrow \text{ L } (-\,0.903) \\ {\text{ H }}-29\rightarrow \text{ L } (-\,0.213) \end{array} \right.$$	–	–	–	–
S$_4$			2.07	600	$$\left\{ \begin{array}{l} {\text{ H }}-11\rightarrow \text{ L } (-\,0.682) \\ {\text{ H }}-4\rightarrow \text{ L } (-\,0.519) \end{array} \right.$$	24	4.73	262	0.0000
S$_5$	2.27	546	2.24	553	$$\left\{ \begin{array}{l} {\text{ H }}-3\rightarrow \text{ L } (-\,0.739) \\ {\text{ H }}-9\rightarrow \text{ L } (-\,0.463) \end{array} \right.$$	3	3.83	324	0.0000
S$_6$	2.41	515	2.41	515	$$\left\{ \begin{array}{l} {\text{ H }}-31\rightarrow \text{ L } (-\,0.651) \\ \beta {\text{ H }}\rightarrow \beta \text{ L } (-\,0.424) \end{array} \right.$$	2	3.75	331	0.0495
S$_7$	2.54	488	2.57	482	$$\left\{ \begin{array}{l} {\text{ H }}\rightarrow \text{ L+12 } (-\,0.651) \\ \text{ H }\rightarrow \text{ L+13 } (-\,0.424) \end{array} \right.$$	1	3.75	331	0.0000

Main transitions of spin-flip LC-TDBLYP excitations are also shown with the coefficients of the response functions in parentheses, in which the notations H and L indicate HOMO and LUMO. The oscillator strengths (*f*) of TD$\omega$B97XD are also listed. The presumed quintet (TT) excitation energy is also appended for the dimer in square brackets.

^a^Experimental results in Ref.^52^.

**Figure 6:**
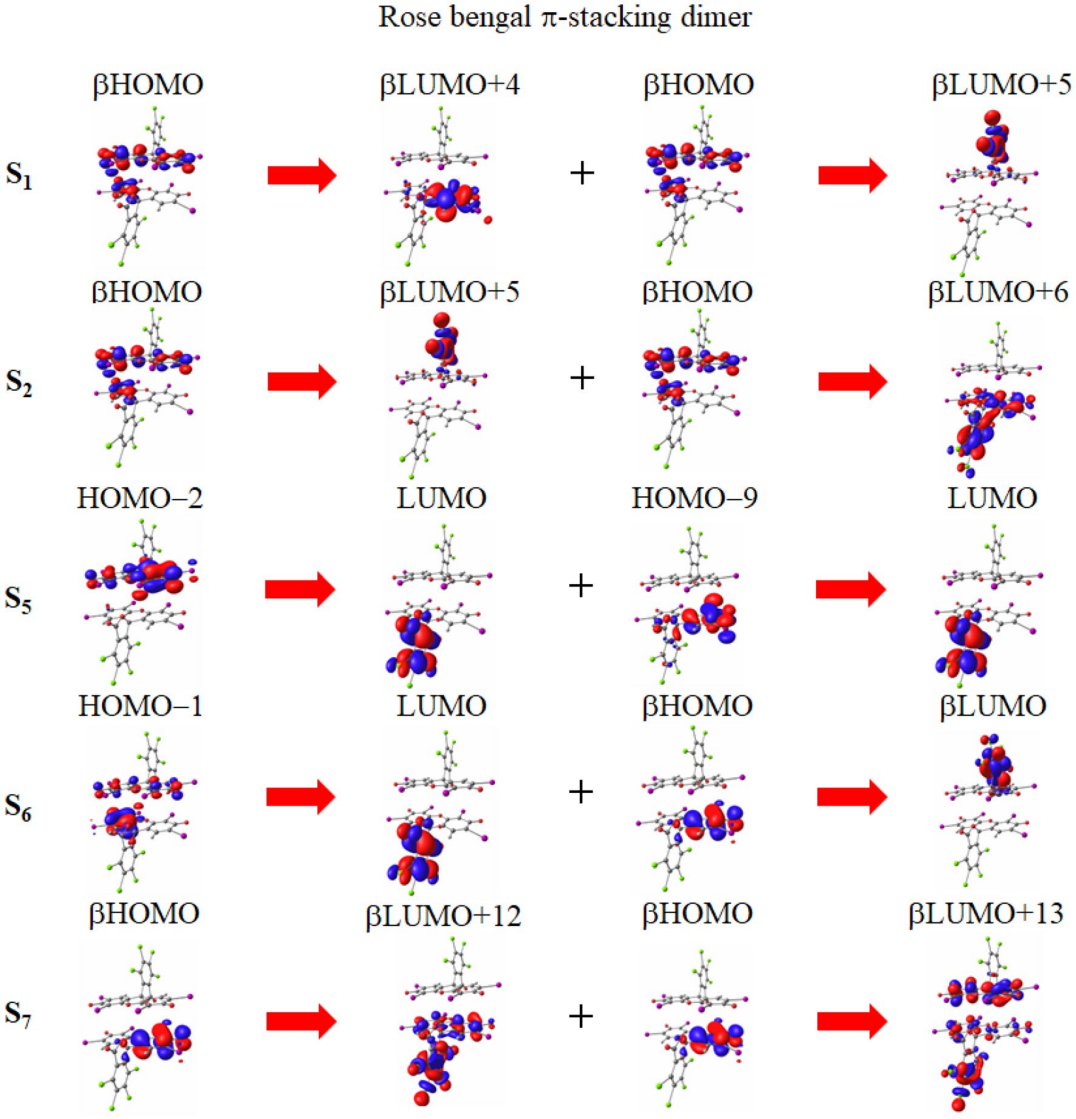
Molecular orbital images corresponding to the main transitions of the S$_1$, S$_2$, and main peak S$_5$ through S$_7$ excitations for the $\pi$-stacking dimer of rose bengal divalent anion molecule in water solvent. The transitions are given in spin-flip TD$\omega$B97XD/cc-pVTZ calculations, while the molecular orbital images are obtained in $\omega$B97XD/cc-pVTZ calculations.

As the table shows, the spin-flip method dramatically decreases the excitation energies to approximately half of their values, indicating the significance of the double excitation effect, similar to that observed in the monomer. Consequently, the correspondence between the excitations of spin-flip LC-TDBLYP and LC-TDBLYP remains controversial for the dimer. However, putting this aside, the main-peak excitation energies are found to be very accurate, with errors of less than 0.03 eV. The spin-flip LC-TDBLYP evaluation of the S$_1$–T$_1$ excitation energy gap remains consistent at 0.56 eV, identical to that of the monomer. Considering the spin-forbidden nature, the ISC between the S$_1$ and T$_1$ states is, therefore, not particularly favorable for the $\pi$-stacking dimer. However, rose bengal dimer exhibits unique characteristics in its singlet excitations. In Fig. 6, the main transitions are illustrated for the S$_1$, S$_2$, and main-peak S$_5$ through S$_7$ excitations.

The figure clearly shows that all the main transitions of these excitations have long-range charge transfer characters. Notably, the main transition of the S$_1$ excitation involves long-range charge transfer between the monomers. Furthermore, similar to the monomer, the S$_1$ excitation contains substantial $n\pi ^*$ transitions, suggesting large spin-orbit coupling with closely-lying triplet excitations. In fact, there are numerous low-lying triplet excitations close to the singlet excitation for rose bengal (see Table S3 in the supporting information). This indicates that ISC is likely to proceed for the rose bengal dimer. In the case of SF, the (TT)$_1$ excitation energy is expected to be double that of the T$_1$ state, i.e., approximately 1.34 eV, according to the spin-flip LC-TDBLYP calculation. Although this energy is higher than the S$_1$ excitation energy, implying an unfavorable deexcitation from the S$_1$ to (TT)$_1$ states, it should be noted that deexcitation to the singlet $^1$(TT) states is a spin-allowed transition and can proceed from the S$_2$ and other singlet states during their lifetimes. In fact, the table shows that the S$_2$ excitation is only 0.08 eV above the (TT)$_1$ excitation, indicating rapid transitions between these states. Consequently, it is expected that SF smoothly takes place through the spin-allowed deexcitation from the S$_2$ to (TT)$_1$ states, playing a significant role in the triplet generation of the heavy atom-containing rose bengal.

### Verification of triplet generation mechanisms in comparison with experimental findings

Finally, we validate the results obtained through the above calculations by comparing them with experimental findings. Conclusively, the calculations demonstrate that all the calculated photosensitizers meet the conditions for SF to proceed: 1. Near-degenerate low-lying S and (TT) excitations with a significant S-T energy gap, and 2. Moderate $\pi$-stacking energy of chromophores. On the other hand, there is partial satisfaction of the El-Sayed rule for benzophenone, BODIPY and methylene blue, while rose bengal shows substantial satisfaction. This suggests that ISC may simultaneously proceed at a very slow rate. For heavy atom-free organic molecules, SF, being a spin-allowed process, is significantly faster than spin-forbidden ISC. Note that after $\pi$-stacking formation, the subsequent SF process proceeds so rapidly that it does not necessitate a discussion of dynamics on the potential energy surface involving nuclear motion. Therefore, SF is expected to preferentially occur in the presence of incident light with the energy of the singlet excitation transitioning to the (TT) excitation.

As an experimental verification of the above conclusions, let us examine the presence of TT annihilation. TT annihilation is the reverse process of SF and results in delayed fluorescence when it occurs:

3 $$\begin{aligned} 2\text{ T } \rightarrow ^5\text{(TT) } \rightarrow ^1\text{(TT) } \rightarrow \text{ S}_0 + \text{ h }\nu . \end{aligned}$$

If this reverse process proceeds, it is natural to expect the forward process as well. Observing delayed fluorescence has become one of the experimental methods to confirm SF. Additionally, a commonly cited reason for the absence of SF is the concentration of chromophores. In other words, at low chromophore concentrations, there are insufficient $\pi$-stackings for SF to occur. However, if TT annihilation takes place, this reasoning no longer holds. This is because TT annihilation progresses through the aggregation-mediated $\pi$-stacking, which is the same requirement for SF. If there is no concentration at which SF can occur, TT annihilation will not occur either. Upon investigating TT annihilation or delayed fluorescence experiments for the photosensitizers targeted in this study, it is found that observations have been reported for all of them^53–59^. These experimental results strongly support the conclusion of this study that triplet generations in heavy atom-free organic molecules preferentially proceed through SF.

Next, let us verify the occurrence of SF in the triplet generation mechanism of each photosensitizer by comparing it with experimental results.

For benzophenone, experimental studies have suggested that the triplet generation proceeds even in isolation. This is because benzophenone undergoes a photochemical reaction in the triplet state upon photoirradiation corresponding to the singlet (S$_1$) excitation of the monomer (360 nm)^60^. This result aligns with computational findings indicating slow progression of ISC in benzophenone. Concerning the S$_8$ excitation (205 nm), which is expected to induce SF, the maximum absorption peak is observed at this wavelength in the UV–Vis spectrum^61^, but there is yet no study specifically targeting this absorption. However, an experimental study using picosecond time-resolved absorption spectroscopy on benzophenone single crystals suggests the occurrence of SF^62^. We anticipate that this process will be experimentally confirmed in the near future.

There are a lot of experimental studies on BODIPY derivatives, with some suggesting the progression of SF. For instance, as discussed in “Introduction” section, we theoretically validated the triplet generation mechanism of TMBODIPY derivatives, which is experimentally suggested by Montero and coworkers^19^, and provided strong support for the occurrence of SF^18^. According to the UV–Vis spectrum of BODIPY^55^, the absorption peaks at 500 nm, with a relatively low and broad absorption band around 375 nm. As the absorption peak near 400 nm, corresponding to the $^1$(TT) excitation, is not prominent assuming dimer formation, SF is likely to proceed through transitions from the S$_3$ excitation (375 nm). In the case of TMBODIPY derivatives, where two distinct triplet generations with different rates are clearly observed^19^, simultaneous ISC is anticipated for BODIPY as well, in line with the calculated results.

The triplet generation mechanism of methylene blue has been experimentally analyzed in detail using broadband transient absorption and two-dimensional electronic spectroscopy with a time resolution of about 10 fs^49^. According to the analysis by Scholes and coworkers^49^, the photodynamics of methylene blue in the monomer involve vibrational relaxation (1–10 ps), followed by ISC and internal conversion (approximately 370 ps), resulting in triplet formation. In the dimer, after generating a new singlet excitation, it disappears within about 10 ps, relaxing to the ground state in 3–4 ps, with a faster relaxation observed at higher concentrations. Surprisingly, these experimental findings align completely with the triplet generation mechanism revealed in this study, except for the final stage where the dimer only relaxes to the ground state. According to the calculated results, following the generation of the singlet (TT) excitation, it undergoes conversion to the quintet state, and subsequently, the dimer either splits into individual triplet monomers or relaxes to the ground state. The apparent discrepancy arises because the experimental analysis does not consider SF and only tracks the behavior of the dimer, assuming triplet generation solely through ISC. In other words, the observation does not encompass either the spin conversion of the (TT) excitation or its separation from the dimer into individual triplet monomers. Therefore, we consider that this experimental analysis strongly supports the triplet generation mechanism proposed in this study.

The triplet generation of rose bengal has also been experimentally investigated using time-resolved photoelectron spectroscopy in combination with transient absorption spectroscopy with a time resolution of about 50 fs in the gas phase^50^. Kappes and coworkers^50^ observed a rapid oscillatory decay of singlet excitation-related probe signals on a time scale of 0–4 ps after the absorption of the maximum absorption peak (538 nm) by the isolated rose bengal divalent anion in the gas phase. They attributed this phenomenon to structural relaxation via a torsional oscillation of the benzyl moiety against the xanthene framework. However, considering our calculation results, we propose that in the gas phase, the singlet (TT) excitation of divalent rose bengal anion is converted to the quintet (TT) one, and then leads to the split into individual triplets or relaxation to the ground state. Additionally, they showed that the time scale for triplet generation significantly increases for the rose bengal monovalent anion with one attached proton. This is expected to result from the change in spin state, preventing the formation of the rose bengal singlet (TT) excitation and, consequently, halting the progression of SF. These experimental findings strongly support our results suggesting rapid SF to generate triplet states even in rose bengal containing heavy iodine atoms.

## Conclusions

In this study, we have theoretically explored the triplet generation mechanism of representative four organic photosensitizers, i.e., benzophenone, BODIPY, methylene blue and rose bengal, focusing on the feasibility of singlet fission (SF), using the spin-flip long-range corrected density functional theory calculation, which incorporates both long-range exchange and double excitation correlation effects and has been confirmed to reproduce accurate excitation energies of large systems. We have, consequently, concluded that SF through the $\pi$-stacking plays a major role in the triplet generations of all these photosensitizers. This is supported by the verification that these photosensitizers meet both conditions: 1. Near-degenerate low-lying S and (TT)$_1$ excitations with a significant S-T energy gap, and 2. Moderate $\pi$-stacking energy of chromophores, which is higher than but not far from the solvation energy, allowing the dissociation to generate triplet-state chromophores.

This study has theoretically revealed that both fast SF and slow intersystem crossing (ISC) occur simultaneously for the triplet generation of all these photosensitizers. For benzophenone, we found that the monomer exhibits the $n\pi ^*$ character in the S$_1$ excitation, allowing the ISC to proceed slowly. Conversely, the $\pi$-stacking dimer displays the main-peak S$_8$ excitation slightly above the (TT)$_1$ excitation, indicating that SF primarily generates triplet states through the formation of the $\pi$-stacking dimer. Similarly, we found that the triplet states of BODIPY are predominantly generated by SF through $\pi$-stacking, though ISC also proceeds very slowly from the S$_1$ excitation. This is evidenced by the main-peak S$_3$ excitation being close to the (TT)$_1$ excitation for the $\pi$-stacking dimer, despite the S$_1$ excitation of the monomer mainly consisting of $\pi \pi ^*$ transitions. For the monovalent cation of methylene blue, the S$_1$ excitation of the monomer exhibits a $\pi \pi ^*$ character with a slight $n\pi ^*$ component, suggesting a very low ISC rate. However, the closely-lying S$_2$ and (TT)$_1$ excitations are expected to initiate SF. In contrast, our findings for the divalent anion of rose bengal, which includes heavy iodine groups, reveal that all S$_1$ and main-peak S$_4$ through S$_6$ excitations feature $n\pi ^*$ characters and have closely-lying triplet excitations. Though this result appears to suggest that ISC proceeds preferentially, we conclude that for the rose bengal divalent anion, spin-allowed SF proceeds even more rapidly, because the (TT)$_1$ excitation is located just below the maximum peak of the S$_2$ excitation. All these mechanisms are robustly supported by comparing them with experimental studies.

We hope that these findings will contribute to the future development of organic photosensitizers.

### Supplementary Information

Supplementary Information.

## Data Availability

All data generated or analyzed during this study are included in this published article and its supplementary information files but are available from the corresponding author upon reasonable request.
