# Supplementary material for: Singlet fission initiating organic photosensitizations
Source: Sci Rep. 2024 Jan 8;14:829. doi: 10.1038/s41598-023-50860-4 (PMC10774408; doi:10.1038/s41598-023-50860-4)
Supplement: Supplementary file 1 — Supplementary Information. [file 41598_2023_50860_MOESM1_ESM.pdf]

Supporting Information

Singlet fission initiating organic photosensitizations

Takao Tsuneda[*,a,b] and Tetsuya Taketsugu[a,c]

[a] Department of Chemistry, Faculty of Science, Hokkaido University, Sapporo 060-0810, Japan

[b] Graduate School of Science Technology and Innovation, Kobe University, Nada-ku, Kobe, Hyogo 657-8501, Japan

[c] Institute for Chemical Reaction Design and Discovery (WPI-ICReDD), Hokkaido University, Sapporo 001-0021, Japan

* Corresponding author: tsuneda@phoenix.kobe-u.ac.jp

Fig. S1. The optimized structures of benzophenone monomer and π-stacking dimer adsorbing isopropyl alcohol molecules, which are calculated using ωB97XD/cc-pVTZ method.

Benzophenone monomer

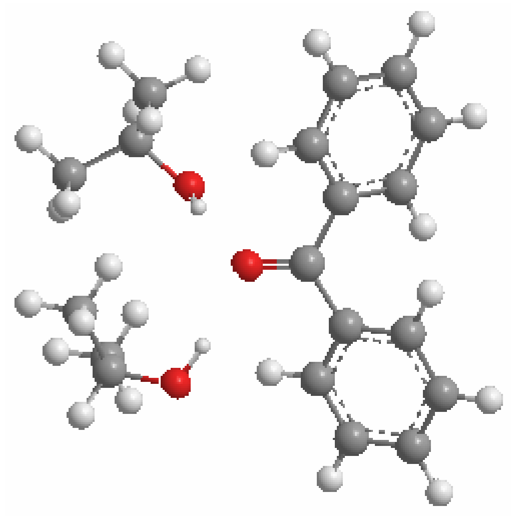

Benzophenone π-stacking dimer

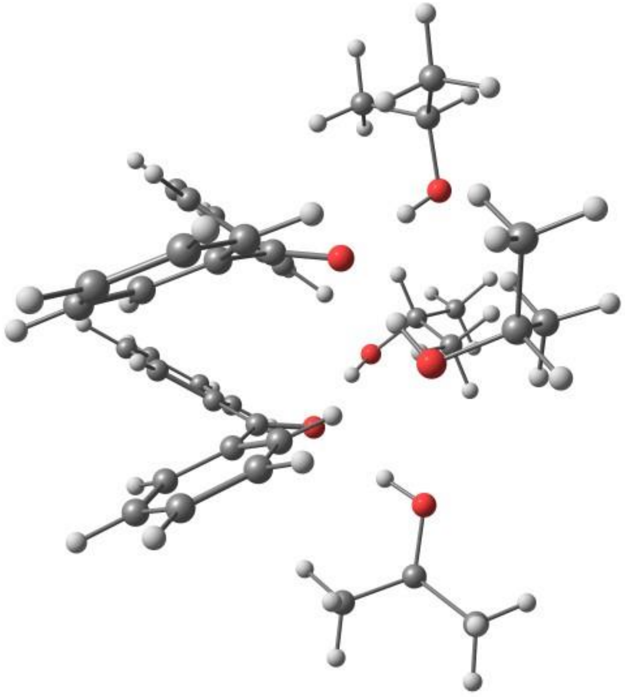

Table S1. The Cartesian coordinates of the optimized π-stacking structures of benzophenone, BODIPY, methylene blue monovalent cation and rose bengal divalent anion, which are calculated using ωB97XD/cc-pVTZ method. The next values of the element names are the corresponding atomic charges.

π-Stacking structure of benzophenone

C	6	-2.2637120	-0.7186490	0.3603420
C	6	-2.8831220	-0.2485730	1.5848870
C	6	-2.2380390	-0.4072370	2.8184720
C	6	-2.8119620	0.0867060	3.9735420
C	6	-4.0410140	0.7253350	3.9133490
C	6	-4.6961360	0.8765520	2.6974700
C	6	-4.1210080	0.4025830	1.5365330
O	8	-2.5264490	-0.1214460	-0.7299160
C	6	-1.3713320	-1.8616560	0.2965250
C	6	-0.3524120	-1.8833260	-0.6586940
C	6	0.4701680	-2.9862550	-0.7824430
C	6	0.2762420	-4.0836940	0.0429240
C	6	-0.7361980	-4.0786520	0.9936240
C	6	-1.5580510	-2.9747770	1.1274680

H	1	-1.2728980	-0.8866470	2.8752910
H	1	-2.2968770	-0.0196670	4.9168870
H	1	-4.4919720	1.1085390	4.8182670
H	1	-5.6568100	1.3689470	2.6572670
H	1	-4.6296270	0.5324820	0.5943130
H	1	-0.1862520	-1.0339700	-1.3057450
H	1	1.2459920	-2.9084690	-1.5293820
H	1	0.9176570	-4.9497130	-0.0508100
H	1	-0.8904600	-4.9406760	1.6263390
H	1	-2.3643570	-2.9869050	1.8447680
C	6	1.9414980	0.4135100	0.9332500
C	6	0.9614840	1.2837660	1.5469450
C	6	0.9870120	1.5716950	2.9169380
C	6	0.0883680	2.4842080	3.4391910
C	6	-0.8298870	3.1099780	2.6066610
C	6	-0.8568690	2.8308990	1.2457620
C	6	0.0355200	1.9212210	0.7227690
O	8	2.2931290	0.7054240	-0.2544330
C	6	2.5403960	-0.7309620	1.5874160

C	6	3.8188270	-1.1579370	1.2069790
C	6	4.3735370	-2.2770780	1.7921020
C	6	3.6550490	-2.9935030	2.7405600
C	6	2.3838160	-2.5863580	3.1189210
C	6	1.8310230	-1.4553230	2.5537530
H	1	1.7284610	1.1162520	3.5557290
H	1	0.1140620	2.7191780	4.4931530
H	1	-1.5255560	3.8274710	3.0194680
H	1	-1.5504990	3.3302390	0.5861490
H	1	0.0125200	1.6824810	-0.3265190
H	1	4.3730620	-0.6027390	0.4667320
H	1	5.3634840	-2.5991500	1.5041900
H	1	4.0887660	-3.8776800	3.1867860
H	1	1.8227070	-3.1545110	3.8460690
H	1	0.8345970	-1.1490510	2.8350630
C	6	-3.6523940	-1.8803590	-3.2334960
C	6	-3.5715080	-2.6307040	-4.5477310
C	6	-3.9507860	-2.8174660	-2.0665630
O	8	-2.4442460	-1.1858500	-3.0503230

H	1	-4.4964940	-1.1858580	-3.3093140
H	1	-3.3873630	-1.9411350	-5.3618990
H	1	-4.4911440	-3.1667960	-4.7460690
H	1	-2.7529850	-3.3414370	-4.5047520
H	1	-4.0795010	-2.2574880	-1.1478810
H	1	-4.8544720	-3.3907400	-2.2410170
H	1	-3.1175830	-3.5014410	-1.9361740
H	1	-2.4488720	-0.7889570	-2.0708860
C	6	-2.8936200	3.0530670	-1.8937810
C	6	-1.8979890	2.3201890	-2.7863050
C	6	-4.0695540	3.5548730	-2.7211310
O	8	-3.3614320	2.2781970	-0.8242220
H	1	-2.3898920	3.9288490	-1.4728020
H	1	-1.4466760	3.0182450	-3.4815750
H	1	-2.3876340	1.5282450	-3.3457970
H	1	-1.0966160	1.8637730	-2.2225770
H	1	-4.5954150	2.7069720	-3.1471100
H	1	-4.7555850	4.1071220	-2.0914810
H	1	-3.7398170	4.2006340	-3.5270820

H	1	-2.9854800	1.2829280	-0.8440610
C	6	3.6085560	-1.3006070	-2.7874480
C	6	4.2542330	0.0441310	-3.1195880
C	6	4.6503470	-2.2357420	-2.1681290
O	8	2.5155970	-1.1082590	-1.9502070
H	1	3.2524510	-1.7500710	-3.7127750
H	1	3.5076910	0.7266790	-3.5069430
H	1	5.0391860	-0.0717360	-3.8584530
H	1	4.6831780	0.4913590	-2.2243810
H	1	5.3029830	-2.6350100	-2.9369450
H	1	5.2627360	-1.7222840	-1.4323050
H	1	4.1445510	-3.0529030	-1.6709590
H	1	2.6467870	-0.3291390	-1.2419240
C	6	3.6872710	3.6744900	-0.8252160
C	6	4.5374890	2.8325510	0.1199120
C	6	4.4244170	3.9168300	-2.1356620
O	8	2.4265010	3.1075630	-1.0788920
H	1	3.5283140	4.6417910	-0.3436260
H	1	5.4997800	3.2984980	0.2920620

H	1	4.7022830	1.8484140	-0.3141240
H	1	4.0398900	2.7137430	1.0758160
H	1	4.5431230	2.9716270	-2.6604740
H	1	3.8553140	4.5918810	-2.7624760
H	1	5.4027710	4.3493110	-1.9605340
H	1	2.3757360	2.1211180	-0.7021990

π-Stacking structure of BODIPY

B	5	-2.2799710	1.5066600	0.4483510
N	7	-2.7487210	0.1382840	-0.1161700
C	6	-3.8230660	-0.5574570	0.2547710
C	6	-3.9187590	-1.7400930	-0.4890980
C	6	-2.8380570	-1.7555770	-1.3492380
C	6	-2.1065360	-0.5781300	-1.1144090
C	6	-0.9475610	-0.0991430	-1.6959000
C	6	-0.4034180	1.1164390	-1.3242210
N	7	-1.0031380	1.8937540	-0.3452750
C	6	-0.2835260	3.0093090	-0.2176710

C	6	0.7998250	2.9940170	-1.1037150
C	6	0.7297950	1.8008250	-1.7963380
F	9	-1.9934800	1.3982040	1.8098370
F	9	-3.2714770	2.4683800	0.2644900
B	5	2.2800610	-1.5067650	-0.4481870
N	7	2.7486190	-0.1382070	0.1159940
C	6	3.8227180	0.5577630	-0.2553530
C	6	3.9184310	1.7403700	0.4885250
C	6	2.8380870	1.7555670	1.3491410
C	6	2.1067220	0.5780080	1.1145450
C	6	0.9477640	0.0990080	1.6961320
C	6	0.4033830	-1.1163650	1.3242420
N	7	1.0030650	-1.8936870	0.3452440
C	6	0.2834590	-3.0092040	0.2176490
C	6	-0.7998930	-2.9939330	1.1037430
C	6	-0.7298640	-1.8007750	1.7963530
F	9	3.2715120	-2.4684590	-0.2638010
F	9	1.9939160	-1.3986900	-1.8097860
H	1	-4.4791570	-0.1922920	1.0282100

H	1	-4.6906200	-2.4829950	-0.3895140
H	1	-2.5756170	-2.5136160	-2.0679300
H	1	-0.4507560	-0.6863490	-2.4542500
H	1	-0.5602520	3.7679630	0.4963360
H	1	1.5437100	3.7652070	-1.1978890
H	1	1.4042580	1.4295500	-2.5490340
H	1	4.4787290	0.1925620	-1.0288580
H	1	4.6899950	2.4835330	0.3885960
H	1	2.5757750	2.5135770	2.0679120
H	1	0.4511430	0.6861330	2.4546660
H	1	0.5602330	-3.7679480	-0.4962450
H	1	-1.5436700	-3.7652150	1.1980160
H	1	-1.4042650	-1.4292870	2.5490100

π-Stacking structure of methylene blue monovalent cation dimer (a)

C	6	5.1593620	-1.7430530	1.9701200
N	7	4.9874870	-1.6676490	0.5266610
C	6	6.1729510	-1.7273640	-0.3174840
C	6	3.7609650	-1.6986850	-0.0109750
C	6	3.5846610	-1.6870110	-1.4331760
C	6	2.3469080	-1.6913090	-1.9763600
C	6	1.1698270	-1.7214750	-1.1747130
C	6	1.3552490	-1.7561540	0.2418210
C	6	2.6095080	-1.7408130	0.8019100
S	16	-0.0018630	-1.8319510	1.3085870
C	6	-1.3568530	-1.7564980	0.2390170
C	6	-2.6123030	-1.7423120	0.7965120
C	6	-3.7620130	-1.6981310	-0.0187060
N	7	-4.9896230	-1.6678980	0.5164780
C	6	-6.1734390	-1.7189410	-0.3305290
C	6	-5.1645360	-1.7489170	1.9592700
C	6	-3.5827410	-1.6832570	-1.4404890
C	6	-2.3438370	-1.6879540	-1.9810950

C	6	-1.1684880	-1.7203860	-1.1770430
N	7	0.0013350	-1.7146990	-1.7995110
H	1	6.2131020	-1.6477230	2.2061410
H	1	4.6239020	-0.9347360	2.4681360
H	1	4.8013770	-2.6966850	2.3620410
H	1	6.2239630	-2.6690200	-0.8652360
H	1	7.0537800	-1.6450890	0.3088570
H	1	6.1892710	-0.9040130	-1.0303860
H	1	4.4437290	-1.6768440	-2.0835450
H	1	2.2124940	-1.6730360	-3.0483190
H	1	2.7013060	-1.7656500	1.8757330
H	1	-2.7063940	-1.7685240	1.8701130
H	1	-6.1849910	-0.8915730	-1.0389660
H	1	-6.2266430	-2.6574270	-0.8835050
H	1	-7.0553650	-1.6369750	0.2943010
H	1	-4.8048240	-2.7029790	2.3484320
H	1	-6.2190600	-1.6573590	2.1932550
H	1	-4.6323340	-0.9410290	2.4615640
H	1	-4.4404520	-1.6710820	-2.0926260

H	1	-2.2071600	-1.6682770	-3.0527410
C	6	5.1603330	1.7440870	-1.9700990
N	7	4.9884440	1.6681610	-0.5266650
C	6	6.1739950	1.7206340	0.3177410
C	6	3.7620250	1.6989820	0.0109410
C	6	3.5856870	1.6856510	1.4331270
C	6	2.3479120	1.6903830	1.9762650
C	6	1.1709070	1.7220060	1.1745860
C	6	1.3563500	1.7576780	-0.2418810
C	6	2.6105860	1.7423050	-0.8019840
S	16	-0.0008190	1.8344730	-1.3086420
C	6	-1.3558250	1.7580030	-0.2391200
C	6	-2.6112380	1.7436330	-0.7966080
C	6	-3.7609520	1.6986030	0.0186340
N	7	-4.9885470	1.6684340	-0.5163790
C	6	-6.1721920	1.7176390	0.3309510
C	6	-5.1637810	1.7479540	-1.9592120
C	6	-3.5816560	1.6828190	1.4404210
C	6	-2.3427730	1.6878680	1.9810140

C	6	-1.1674140	1.7212260	1.1769640
N	7	0.0023640	1.7151250	1.7993980
H	1	6.2144170	1.6524760	-2.2059140
H	1	4.7991430	2.6965190	-2.3618710
H	1	4.6275860	0.9340860	-2.4684120
H	1	7.0546720	1.6406630	-0.3090810
H	1	6.1888030	0.8930920	1.0259390
H	1	6.2268190	2.6589620	0.8710130
H	1	4.4447890	1.6739980	2.0834330
H	1	2.2134330	1.6710620	3.0481970
H	1	2.7024470	1.7673610	-1.8758030
H	1	-2.7053750	1.7704190	-1.8701950
H	1	-6.2236390	2.6536320	0.8882370
H	1	-6.1853480	0.8869600	1.0355720
H	1	-7.0543190	1.6404200	-0.2941820
H	1	-6.2181490	1.6542370	-2.1930170
H	1	-4.8059590	2.7024590	-2.3490450
H	1	-4.6300170	0.9408750	-2.4611550
H	1	-4.4394070	1.6694010	2.0924850

H	1	-2.2060310	1.6674460	3.0526390

π-Stacking structure of methylene blue monovalent cation dimer (b)

C	6	4.2282680	3.1614310	-1.1147590
N	7	4.0918780	2.3771370	0.1025930
C	6	5.2783540	2.1452950	0.9131840
C	6	2.8799120	2.0013330	0.5320960
C	6	2.7278230	1.2950330	1.7692680
C	6	1.5078910	0.8930310	2.1894540
C	6	0.3237630	1.1643730	1.4462560
C	6	0.4808160	1.8864640	0.2247810
C	6	1.7186830	2.2887610	-0.2164820
S	16	-0.8908100	2.2684320	-0.7561990
C	6	-2.2185460	1.6114050	0.1324010
C	6	-3.4799370	1.7602290	-0.3926500
C	6	-4.6086210	1.2532920	0.2865170
N	7	-5.8385250	1.3778890	-0.2250530
C	6	-7.0079550	0.9195400	0.5140480
C	6	-6.0472370	2.0933380	-1.4751830
C	6	-4.4021930	0.5977030	1.5452740

C	6	-3.1579390	0.4448910	2.0482840
C	6	-2.0029360	0.9370820	1.3740500
N	7	-0.8277050	0.7402430	1.9505640
H	1	5.2809320	3.3314110	-1.3104400
H	1	3.8016930	2.6338310	-1.9685430
H	1	3.7339450	4.1295900	-1.0188960
H	1	5.2738420	2.7608840	1.8138710
H	1	6.1575620	2.3912050	0.3281500
H	1	5.3519420	1.0989460	1.2036550
H	1	3.5896360	1.0733520	2.3761860
H	1	1.3934520	0.3498440	3.1165530
H	1	1.7905790	2.8304110	-1.1460020
H	1	-3.5972890	2.2820700	-1.3288930
H	1	-6.9410980	-0.1439570	0.7376050
H	1	-7.1281020	1.4720210	1.4465730
H	1	-7.8900720	1.0775300	-0.0951960
H	1	-5.7531870	3.1401120	-1.3837260
H	1	-7.0984410	2.0516640	-1.7348180
H	1	-5.4799020	1.6372430	-2.2863240

H	1	-5.2445820	0.2226880	2.1030620
H	1	-2.9996790	-0.0577540	2.9914330
C	6	5.9335280	-0.1950520	-2.1190970
N	7	5.8032490	-0.8196290	-0.8119950
C	6	7.0140630	-1.1025810	-0.0525780
C	6	4.6027810	-1.2046740	-0.3624360
C	6	4.4728740	-1.8469400	0.9131740
C	6	3.2628040	-2.2385620	1.3688550
C	6	2.0698950	-2.0339380	0.6187580
C	6	2.2071190	-1.3944610	-0.6517060
C	6	3.4331200	-0.9895070	-1.1220130
S	16	0.8270290	-1.1190350	-1.6537740
C	6	-0.4893130	-1.6852970	-0.6877640
C	6	-1.7615850	-1.5452150	-1.1889950
C	6	-2.8776320	-2.0107080	-0.4618920
N	7	-4.1222990	-1.8648390	-0.9322440
C	6	-5.2602730	-2.4267170	-0.2187470
C	6	-4.3523730	-1.2540170	-2.2313800
C	6	-2.6447120	-2.6447990	0.8024390

C	6	-1.3924290	-2.7669230	1.2933740
C	6	-0.2525660	-2.2915670	0.5843450
N	7	0.9315900	-2.4450200	1.1574050
H	1	6.9681820	0.0857560	-2.2762450
H	1	5.6333850	-0.8788550	-2.9146470
H	1	5.3246270	0.7061330	-2.1830680
H	1	6.9829950	-0.6354270	0.9319500
H	1	7.8648450	-0.6982570	-0.5883860
H	1	7.1616370	-2.1759290	0.0695340
H	1	5.3462120	-2.0257490	1.5185610
H	1	3.1623680	-2.7195970	2.3311410
H	1	3.4905030	-0.5105310	-2.0865590
H	1	-1.8950350	-1.0803180	-2.1527320
H	1	-5.1988880	-3.5142810	-0.1662130
H	1	-5.3218510	-2.0289570	0.7939240
H	1	-6.1696420	-2.1606550	-0.7455120
H	1	-3.8968740	-0.2645800	-2.2768040
H	1	-5.4196090	-1.1443760	-2.3873700
H	1	-3.9435550	-1.8662540	-3.0369140

H	1	-3.4736810	-3.0221920	1.3775930
H	1	-1.2163530	-3.2312890	2.2529120

π-Stacking structure of rose bengal divalent anion

O	8	-4.3459460	-0.0002710	3.1598310
C	6	-4.1365140	-0.0009390	1.9833920
O	8	-2.8876870	-0.0012240	1.4751370
C	6	-5.0794770	-0.0016570	0.8399670
C	6	-4.3371720	-0.0019740	-0.3186020
C	6	-4.9490640	-0.0025430	-1.5549290
Cl	17	-4.0076330	-0.0026190	-2.9996720
C	6	-6.3419760	-0.0029830	-1.6035130
Cl	17	-7.1383110	-0.0039010	-3.1262710
C	6	-7.0984780	-0.0026830	-0.4224500
Cl	17	-8.8173070	-0.0032380	-0.5072150
C	6	-6.4638890	-0.0019390	0.8167080
Cl	17	-7.3720360	-0.0013740	2.2761550
C	6	-2.8592230	-0.0013090	0.0337720
C	6	-2.1596230	1.2403080	-0.4463830
C	6	-1.1698520	1.1753930	-1.4132240
C	6	-0.6107850	2.3399540	-1.9346200
I	53	0.7801290	2.2652700	-3.4764060

C	6	-1.0270370	3.5826880	-1.4637550
O	8	-0.4767700	4.6726390	-2.0070320
C	6	-2.0060990	3.6387760	-0.4639130
I	53	-2.6818220	5.4883020	0.2382960
C	6	-2.5623240	2.4815490	0.0211220
O	8	-0.7265920	-0.0001070	-1.9232930
C	6	-1.1683610	-1.1760880	-1.4130250
C	6	-0.6079040	-2.3400480	-1.9342810
I	53	0.7828180	-2.2641940	-3.4762590
C	6	-1.0228060	-3.5832160	-1.4633400
O	8	-0.4711960	-4.6725810	-2.0064060
C	6	-2.0019830	-3.6403580	-0.4636680
I	53	-2.6758090	-5.4907020	0.2382770
C	6	-2.5595810	-2.4837460	0.0212380
C	6	-2.1581370	-1.2420840	-0.4462340
H	1	-0.8183050	5.4690920	-1.5835440
H	1	-3.3332330	2.5362920	0.7756760
H	1	-0.8121480	-5.4693860	-1.5831050
H	1	-3.3306460	-2.5392660	0.7755720

O	8	4.3500180	0.0022810	-3.1586130
C	6	4.1392440	0.0019780	-1.9824260
O	8	2.8897820	0.0010600	-1.4756530
C	6	5.0808510	0.0025530	-0.8379180
C	6	4.3372060	0.0020390	0.3198060
C	6	4.9478010	0.0024210	1.5567840
Cl	17	4.0049580	0.0017570	3.0006080
C	6	6.3406800	0.0033520	1.6068720
Cl	17	7.1353730	0.0039040	3.1304900
C	6	7.0984870	0.0038380	0.4266670
Cl	17	8.8172170	0.0050080	0.5132580
C	6	6.4652330	0.0033990	-0.8131650
Cl	17	7.3749090	0.0039060	-2.2716570
C	6	2.8595940	0.0011190	-0.0342960
C	6	2.1580110	1.2418430	0.4451940
C	6	1.1682640	1.1757600	1.4120460
C	6	0.6077860	2.3396940	1.9333740
I	53	-0.7825270	2.2639720	3.4757650
C	6	1.0224880	3.5828800	1.4622920

O	8	0.4707980	4.6722220	2.0053310
C	6	2.0015680	3.6400990	0.4625310
I	53	2.6751430	5.4904780	-0.2395370
C	6	2.5592990	2.4835340	-0.0223260
O	8	0.7265240	-0.0002610	1.9222260
C	6	1.1697370	-1.1756960	1.4119630
C	6	0.6106630	-2.3403550	1.9331650
I	53	-0.7798600	-2.2661190	3.4753680
C	6	1.0269310	-3.5830120	1.4621010
O	8	0.4766580	-4.6730540	2.0051850
C	6	2.0060260	-3.6389490	0.4622940
I	53	2.6818350	-5.4883640	-0.2401550
C	6	2.5623370	-2.4816600	-0.0224840
C	6	2.1595760	-1.2405000	0.4451590
H	1	0.8116080	5.4690390	1.5819390
H	1	3.3303330	2.5391410	-0.7766820
H	1	0.8182290	-5.4694400	1.5816030
H	1	3.3334060	-2.5362500	-0.7768770

Table S2-1. The correspondences of the calculated Spin-flip LC-TDBLYP and LC-TDBLYP excitation energies Δε (eV) of benzophenone monomer with the oscillator strengths (*f*) of LC-TDBLYP. The calculations are carried out using cc-pVTZ basis sets with CPCM solvent effect of isopropyl alcohol. The values in the next parentheses are the energies in nm for Δε and the coefficients of the TDDFT response function for main transitions.

Exc.	Exp.	Spin-flip LC-TDBLYP		LC-TDBLYP		*f*
		Δε (eV)	Main transitions	Δε (eV)	Main transitions	
T_1		3.20(387)		2.93(423)		
S_1	3.61(343)	4.20(295)	βH→βL(-0.950), βH→βL+7(0.226)	3.94(315)	H-4→L(0.482), H→L(0.463)	0.0012
S_2	4.40(282)	5.02(247)	H-4→L(-0.780), H-8→L(-0.460)	5.12(242)	H-1→L(0.524), H-3→L+2(-0.247)	0.0314
S_3	5.00(248)	5.33(233)	H-3→L (-0.829), H→L (-0.265)	5.17(240)	H-3→L (0.496), H-1→L+2 (-0.270)	0.0286
S_4	5.00(248)	5.37(231)	H-2→L(-0.831), H-1→L(-0.491)	5.32(233)	H-2→L(-0.621), H-3→L(-0.227)	0.5214
S_5		5.67(219)	H→L(0.948), H-3→L(-0.264)	5.52(224)	H→L+1(0.451), H-4→L(-0.431)	0.0701

Table S2-2. The correspondences of the calculated Spin-flip LC-TDBLYP and TDωB97XD excitation energies Δε (eV) of benzophenone π-stacking dimer with the oscillator strengths (*f*) of TDωB97XD. The calculations are carried out using cc-pVTZ basis sets with CPCM solvent effect of isopropyl alcohol. The values in the next parentheses are the energies in nm for Δε and the coefficients of the TDDFT response function for main transitions.

Exc.	Exp.	Spin-flip LC-TDBLYP		TDωB97XD		*f*
		Δε (eV)	Main transitions	Δε (eV)	Main transitions	
T_1		3.22(386)		2.95(420)		
$(TT)_1$				5.68(218)		
S_1	3.61(343)	4.21(294)	βH→βL+1(-0.929)	5.00(248)	H-5→L(0.364), H→L+1(0.311)	0.0096
S_2	4.40(282)	5.01(247)	H-9→L(0.775), H-18→L(-0.428)	3.99(311)	H-8→L+1(0.364), H-3→L(0.311)	0.0005
S_3	5.00(248)	5.28(235)	H-8→L(0.775), H→L(-0.428)	3.99(311)	H-1→L(0.464), H-7→L+1(0.322)	0.1232
S_4	5.00(248)	5.33(233)	H-6→L(0.775), H-3→L(-0.428)	5.21(238)	H-6→L (0.358), H-4→L+1(0.320)	0.5556
S_5		5.62(221)	H-4→L(0.775), H→L(-0.428)	5.14(241)	H-2→L+1(0.361), H-4→L+1(0.315)	0.0634

S_6		5.72(217)	H-5→L(0.775)	5.88(211)	H-3→L(0.415),<br>H→L+1(0.285)	0.0533
S_7		6.04(205)	βH→βL+3(0.775),<br>βH→βL+2(-0.428)	6.23(199)	H-1→L+1(0.297),<br>H→L+3(0.260)	0.0077
S_8	6.04(205)	6.27(198)	βH→βL (-0.936)	5.01(247)	H→L (0.340),<br>H-4→L (-0.322)	0.0248
S_9		6.34(195)	βH→βL+5 (0.700)	5.35(232)	H→L (0.370),<br>H-8→L (-0.267)	0.0956

Table S2-3. The correspondences of the calculated Spin-flip LC-TDBLYP and LC-TDBLYP excitation energies Δε (eV) of BODIPY monomer with the oscillator strengths (*f*) of LC-TDBLYP. The calculations are carried out using cc-pVTZ basis sets with CPCM solvent effect of water. The values in the next parentheses are the energies in nm for Δε and the coefficients of the TDDFT response function for main transitions.

Exc.	Exp.	Spin-flip LC-TDBLYP		LC-TDBLYP		*f*
		Δε (eV)	Main transitions	Δε (eV)	Main transitions	
T_1		1.62(768)		1.43(867)		
S_1	2.48(500)	2.27(546)	H→L(-0.697), βH→βL(-0.682)	2.93(423)	H→L(-0.694)	0.6063
T_2	3.31(375)	3.30(376)	H-1→L(0.776), H→L(-0.472)	-	-	-
S_2		3.61(343)	H-2→L(0.926)	4.46(277)	H-2→L(0.697)	0.0663

Table S2-4. The correspondences of the calculated Spin-flip LC-TDBLYP and TDωB97XD excitation energies Δε (eV) of BODIPY π-stacking dimer with the oscillator strengths (*f*) of TDωB97XD. The calculations are carried out using cc-pVTZ basis sets with CPCM solvent effect of water. The values in the next parentheses are the energies in nm for Δε and the coefficients of the TDDFT response function for main transitions.

Exc.	Exp.	Spin-flip LC-TDBLYP		TDωB97XD		*f*
		Δε (eV)	Main transitions	Δε (eV)	Main transitions	
T_1		1.63(759)		1.43(867)		
$(TT)_1$				2.72(456)		
S_1	2.48(500)	2.29(542)	H→L+1(-0.661),<br>H-1→L(-0.660)	3.10(399)	H-1→L(0.526),<br>H→L+1(-0.446)	1.0845
T_2		3.30(376)	H-4→L(-0.584),<br>H-1→L(-0.485)	-	-	-
S_2		3.39(366)	H→L(-0.763),<br>H-5→L(-0.440)	2.74(452)	H→L(0.591),<br>H-1→L+1(-0.372)	0.0000
S_3	3.31(375)	3.46(358)	H^2→L^2(0.808),<br>βH→βL(0.410)	3.90(318)	H-1→L+1(0.574),<br>H→L(0.371)	0.2236

Table S2-5. The correspondences of the calculated Spin-flip LC-TDBLYP and LC-TDBLYP excitation energies Δε (eV) of methylene blue monovalent cation monomer with the oscillator strengths (*f*) of LC-TDBLYP. The calculations are carried out using cc-pVTZ basis sets with CPCM solvent effect of water. The values in the next parentheses are the energies in nm for Δε and the coefficients of the TDDFT response function for main transitions. For the S_3 excitation, no corresponding excitations is found in the 20 calculated excitations of LC-TDBLYP.

Exc.	Exp.	Spin-flip LC-TDBLYP		LC-TDBLYP		*f*
		Δε (eV)	Main transitions	Δε (eV)	Main transitions	
T_1		1.21(1021)		1.16(1066)		
S_1	1.87(663)	1.78(698)	βH→βL(-0.705), H→L(-0.682)	2.45(506)	H→L(0.691)	1.1577
T_2		2.59(478)	H-1→L(0.776), H^2→L^2(-0.472)	-	-	-
S_2		3.10(399)	βH→βL(-0.645), H→L(0.644)	-	-	-
S_3		3.22(386)	H-2→L(0.968)	3.04(408)	H→L+1(0.517), H-2→L(0.358)	0.0150

Table S2-6. The correspondences of the calculated Spin-flip LC-TDBLYP and TDωB97XD excitation energies Δε (eV) of methylene blue monovalent cation π-stacking dimer (1) with the oscillator strengths (*f*) of TDωB97XD. The calculations are carried out using cc-pVTZ basis sets with CPCM solvent effect of water. The values in the next parentheses are the energies in nm for Δε and the coefficients of the TDDFT response function for main transitions.

Exc.	Exp.	Spin-flip LC-TDBLYP		TDωB97XD		*f*
		Δε (eV)	Main transitions	Δε (eV)	Main transitions	
T_1		1.21(1021)		1.54(805)		
$(TT)_1$	2.05(606)	[2.04(609)]		2.59(478)		
S_1		1.76(705)	H→L+1(-0.676),<br>H-3→L(-0.524)	2.79(445)	H→L+1(0.614),<br>H-1→L(0.342)	0.1191
T_2		2.54(489)	H-2→L(-0.736),<br>H-3→L(-0.588)	-	-	-
S_2		2.81(442)	H→L(-0.697),<br>βH→βL(-0.615)	2.17(571)	H→L(0.628),<br>H-1→L+1(-0.303)	0.0000
T_3		2.84(437)	H^2→L^2(-0.697)	-	-	-
S_3	2.38(520)	2.89(430)	βH→βL(0.709),<br>H→L+1(-0.647)	2.59(478)	H-1→L(0.608),<br>H→L+1(-0.332)	1.7203

Table S2-7. The correspondences of the calculated Spin-flip LC-TDBLYP and TDωB97XD excitation energies Δε (eV) of methylene blue monovalent cation π-stacking dimer (2) with the oscillator strengths (f) of TDωB97XD. The calculations are carried out using cc-pVTZ basis sets with CPCM solvent effect of water. The values in the next parentheses are the energies in nm for Δε and the coefficients of the TDDFT response function for main transitions.

Exc.	Exp.	Spin-flip LC-TDBLYP		TDωB97XD		f
		Δε (eV)	Main transitions	Δε (eV)	Main transitions	
T_1		1.23(1004)		1.44(861)		
$(TT)_1$	2.05(606)	[2.04(609)]		2.49(497)		
S_1		1.79(694)	H→L+1(-0.695), H-2→L(-0.600)	2.14(580)	H-1→L(0.530), H→L+1(-0.433)	0.0017
T_2		2.58(481)	H-3→L(-0.819), H-2→L(-0.398)	-	-	-
T_3		2.64(470)	βH→βL(-0.758), H^2→L^2(-0.611)	-	-	-
S_2	2.38(520)	2.84(437)	H→L(0.930)	2.48(499)	H→L(0.606), H-1→L+1(-0.331)	1.8153
S_3		2.89(430)	H→L+1(-0.641), H-2→L(0.580)	2.58(481)	H→L+1(0.441), H-1→L+1(-0.409)	0.1324

Table S2-8. The correspondences of the calculated Spin-flip LC-TDBLYP and LC-TDBLYP excitation energies Δε (eV) of rose bengal divalent anion monomer with the oscillator strengths (*f*) of LC-TDBLYP. The calculations are carried out using cc-pVTZ basis sets with CPCM solvent effect of water. The values in the next parentheses are the energies in nm for Δε and the coefficients of the TDDFT response function for main transitions. For the S_5 and S_7 excitations, no corresponding excitations is found in the 20 calculated excitations of LC-TDBLYP.

Exc.	Exp.	Spin-flip LC-TDBLYP		LC-TDBLYP		*f*
		Δε (eV)	Main transitions	Δε (eV)	Main transitions	
T_1		0.89(1401)				
S_1		1.45(856)	H→L+2(-0.723), H→L+1(-0.621)	4.74(261)	H→L+1(0.269), H-2→L+3(0.261)	0.0057
S_2		1.68(739)	H→L+3(-0.792), H→L+5(0.413)	4.28(290)	H→L+3(0.436), H-1→L+5 (-0.399)	0.0215
S_3		1.74(714)	H-3→L(-0.884), H-15→L(-0.194)	5.19(239)	H-1→L+1(-0.273), H-1→L+6(0.253)	0.0365
S_4		2.30(539)	H-5→L (-0.616), H-1→L(-0.540)	4.21(294)	H-1→L (0.483), H-1→L+6(-0.239)	0.0788
S_5	2.27(546)	2.72(456)	H-16→L(-0.742), H-10→L(-0.532)	-	-	-

S_6	2.41(515)	2.77(447)	βH→βL(0.930), H-5→L(-0.175)	4.14(300)	H→L(0.593)	0.0714
S_7	2.54(488)	2.97(417)	H→L+7(0.838), H→L+2(0.271)	-	-	-

Table S2-9. The correspondences of the calculated Spin-flip LC-TDBLYP and TDωB97XD excitation energies Δε (eV) of rose bengal divalent anion π-stacking dimer (1) with the oscillator strengths (*f*) of TDωB97XD. The calculations are carried out using cc-pVTZ basis sets with CPCM solvent effect of water. The values in the next parentheses are the energies in nm for Δε and the coefficients of the TDDFT response function for main transitions. For the S_3 excitation, no corresponding excitations is found in the 50 calculated excitations of LC-TDBLYP.

Exc.	Exp.	Spin-flip LC-TDBLYP		TDωB97XD		*f*
		Δε (eV)	Main transitions	Δε (eV)	Main transitions	
T_1		0.67(1859)		2.16(575)		
$(TT)_1$				4.31(288)		
S_1		1.23(1004)	H→L+4(0.812), H→L+5(-0.548)	4.29(289)	H→L+11(0.300), H-1→L+10(-0.281)	0.0000
S_2		1.40(883)	H→L+5(-0.689), H→L+6(0.369)	3.95(314)	H→L+6(0.366), H-1→L+7(0.331)	0.0014
S_3		1.51(819)	H-9→L(-0.903), H-29→L(-0.213)	-	-	-
S_4		2.07(600)	H-11→L (-0.682), H-4→L(-0.519)	4.73(262)	H-20→L+1(0.365), H-16→L(0.247)	0.0000

S_5	2.27(546)	2.24(553)	H-3→L(-0.739), H-9→L(-0.463)	3.83(324)	H-2→L+1(0.460), H-3→L (-0.430)	0.0000
S_6	2.41(515)	2.41(515)	H-31→L(-0.651), βH→βL(-0.424)	3.75(331)	H→L(-0.501), H-1→L+1(-0.450)	0.0495
S_7	2.54(488)	2.57(482)	H→L+12 (-0.651), H→L+13(-0.424)	3.75(331)	H→L+1 (0.500), H-1→L(-0.448)	0.0000

Table S3. The calculated triplet excitation energies of four organic photosensitizer molecules: benzophenone, BODIPY, methylene blue monovalent cation and rose bengal divalent anion for the monomers and dimers. The calculations are performed using LC-TDBLYP (monomers) and ωB97XD (dimers) with cc-pVTZ (except for I) and LanL2DZ (I) basis functions.

	Benzophenone		BODIPY		Methylene blue		Rose bengal	
Exc.	Monomer	Dimer	Monomer	Dimer	Monomer	Dimer	Monomer	Dimer
T_1	2.93	2.95	1.43	2.09	1.16	1.54	3.80	2.16
$(TT)_1$		5.68		2.52		2.59		4.31
T_2	4.19	4.39	3.00	2.31	1.93	2.20	3.91	2.85
T_3	4.71	5.31	3.23	3.77	2.95	2.38	4.49	3.35
T_4	5.30	5.65	3.48	3.90	3.01	2.72	4.52	3.39
T_5	5.39	6.02	5.08	4.06	3.41	2.86	4.63	3.67
T_6	6.01	6.13	5.61	4.20	3.61	3.11	5.12	3.81
T_7	6.29	6.14	5.66	4.29	4.05	3.26	5.38	4.15
T_8	6.35	6.27	6.15	4.39	4.32	3.43	5.43	4.31
